# Piezophototronic gated optofluidic logic computations empowering intrinsic reconfigurable switches

**DOI:** 10.1038/s41467-019-12148-y

**Published:** 2019-09-26

**Authors:** Yuvasree Purusothaman, Nagamalleswara Rao Alluri, Arunkumar Chandrasekhar, Vivekananthan Venkateswaran, Sang-Jae Kim

**Affiliations:** 1 Nanomaterials and System Lab, Department of Mechatronics Engineering, Jeju National University, Jeju, 690756 Republic of Korea; 2 Department of Sensor and Biomedical Technology, School of Electronics Engineering, Vellore Institute of Technology, Vellore, 632014 India

**Keywords:** Electronic devices, Nanofluidics, Nanowires

## Abstract

Optofluidic nano/microsystems have advanced the realization of Boolean circuits, with drastic progression to achieve extensive scale integration of desirable optoelectronics to investigate multiple logic switches. In this context, we demonstrate the optofluidic logic operations with interfacial piezophototronic effect to promote multiple operations of electronic analogues. We report an optofluidic Y-channeled logic device with tunable metal-semiconductor-metal interfaces through mechanically induced strain elements. We investigate the configuration of an OR gate in a semiconductor-piezoelectric zinc oxide nanorod-manipulated optofluidic sensor, and its direct reconfiguration to logic AND through compressive strain-induced (−1%) piezoelectric negative polarizations. The exhibited strategy in optofluidic systems implemented with piezophototronic concept enables direct-on chip working of OR and AND logic with switchable photocurrent under identical analyte. Featured smart intrinsic switching between the Boolean optoelectronic gates (OR↔AND) ultimately reduces the need for cascaded logic circuits to operate multiple logic switches on-a-chip.

## Abstract (web summary)

Designing optofluidic nano/microsystems to realize large-scale Boolean circuits remains a challenge. Here, the authors propose a flexible optofluidic framework to perform binary computations with an integrated piezophototronic mechanism controlling the optofluidic switching of logic gates (PPOF).

## Introduction

Potentially fetched functionalities in lab-on-a-chip or microsystems through optofluidic technology have provided better mitigation in medical diagnostics^1,2^, bio-optical computing^3^, water treatments^4,5^, electronic switches^6^, imaging devices^3,6^, photovoltaic cells^7,8^, bio/chemical sensors, and integrated sensors/actuators^9,10^. Implementation of smart, interactive optofluidic tools for human–machine-interfaced next-generation applications demands an active and adaptable circuit interfaced in electromechanical stimuli with the capability of direct electrical information encoded in logic units^11–14^. Prominent control of semiconductor-based photonic and electronic logic gates has activated the most effective way of utilizing the supremacy of electromechanical–optofluidic integrations^15^. Most of the developed optofluidic systems demonstrate the fundamental concept using biomolecules (chemicals) to switch between ON (‘1’) and OFF (‘0’) within a specified Boolean operator (AND, NAND, NOT, OR, XOR, and NOR) based on photoelectric–hydrodynamic conditions^16,18^. Besides, it requires a wide range of fluidic operands making the system challenging to configure multiple logic gates together using the same analyte (fluid)^17^. Conversely, the mechanical triggering of gates was introduced through piezotronic/piezophototronic strain modulations;^19–21^ however, it has complex circuitry and also restricts the implication of chemical tunability to the sensors.

As a route to break these limitations, we have demonstrated the piezophototronic gated optofluidic system (PPOF) with the interfacing of three metrics such as optical–fluidic–strain functionalities. With induced piezoelectric polarization at semiconductor–metal interfaces, we exhibit optofluidic binary computation of the OR gate and its intrinsic configuration to the logic AND. The principal interfacing of optofluidics with piezophototronic devices is not an easy task and so far lacks reports with proof-of-mechanism experiments. Further, the investigations and results are indeed promising to move from proof-of-concept analysis to ready-to-use intrinsic reconfigurable switching devices exceptional from traditional/conventional nano- and microsystems.

Hence, we report the new insight of synergetic tailoring in optofluidic logic devices to explore dual-modulated switching principles facilitated through effective control of the piezophototronic effect. We implemented the MSM interfacial Y-channel optofluidic logic gate using in situ-grown zinc oxide nanorods (ZnO NR) in the PVDF substrate with three-way coupling among piezoelectric, semiconductor, and photoexcitation properties. The chemical tailoring of volatile organic compounds (VOC) serves as multi-addressable binary operands (‘1’ and ‘0’) to approach a photo-molecularly tuned OR gate operator. Compressive strain (*ε*_c_) of −1 % produces significant deviations in the photocurrent which not only controls between ON and OFF gate operations; indeed, it reconfigures OR to intrinsically switch its property addressing the AND gate (OR↔AND). The integration of optofluidics with the piezoelectric system using a multisource-responsive material enables dual-logic functionality on a single platform providing adaptable switches for interactive optoelectronics.

## Results

### Y-channel optofluidic logic gate design

**Fig. 1:**
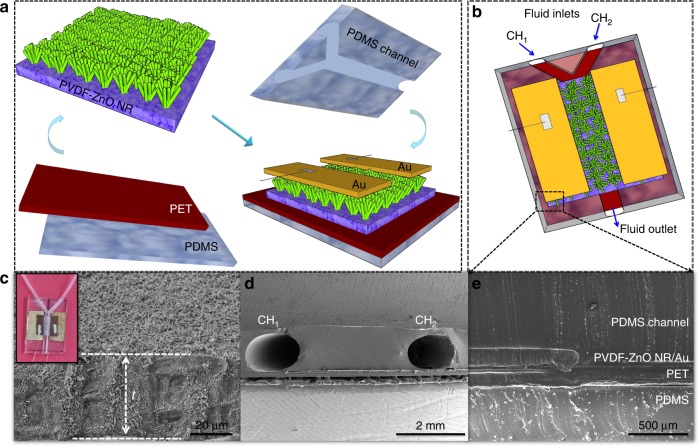
Device design and structure. **a** Schematic representation of the fabrication process with MSM interfaces. **b** Illustration of the Y-channel optofluidic device (Y-OF). **c** Ionized PVDF film grown on ZnO NR with thickness *t*~50 μm (inset: digital photograph of Y-OF). **d** Cross-sectional FESEM images depicting the diameter of the channels (CH_1_/CH_2_) of 1 mm. **e** Layer-by-layer arrangements in developed Y-OF

We have designed an optofluidic sensor with a Y-shaped channel made of the PDMS groove (Y-OF). Figure 1a, b presents the schematic of Y-OF fabrication process and the corresponding digital image is shown in Supplementary Fig. 1. Figure 1c shows the uniformly grown ZnO NR endowed with photoelectric–piezoelectric properties on an ionized PVDF film (thickness, *t* ~ 50 μm) through the in situ method^22^. Supplementary Figs. 2–4 support the confirmation of well-distributed ZnO NR and its compositional mapping. PVDF/ZnO NR acts as the sensing layer to detect ternary sources such as optical, fluidic, and strain promoting piezophototronic switching of optofluidic logic gates. Each of the inlet channels (CH_1_/CH_2_) are of diameter 1 mm (Fig. 1d), and they protrude through PDMS to enable the outward flow of fluids. Figure 1e illustrates the completely packed device with layer-by-layer arrangements such as the PDMS/PET/PVDF–ZnO NR/Au/PDMS channel.

**Fig. 2:**
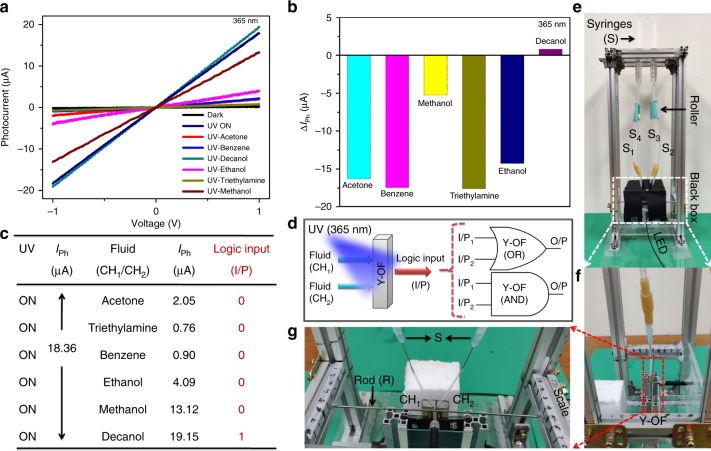
Selections of logic operands. **a** Dark and UV-illuminated *I*–*V* graph with the effect of VOC. **b** Behavior of the change in the photocurrent under the influence of VOC. **c** Table summarizing the suitability of fluidic inputs. **d** Simplified illustration of Y-OF functioning as OR and AND gates. **e** Digital photograph of the experimental setup to perform piezophototronic-based optofluidic switches (PPOF). **f**, **g** Magnified images with a mounted Y-OF device

To perform optofluidic logic gates, we evaluated the suitability of solvents as fluidic sources to determine the input binary states ‘0’ and ‘1’. Analysis of sensing characteristics is performed by using VOC such as triethylamine (T), benzene (B), acetone (A), ethanol (E), decanol (D), and methanol (M). All the solvents are used with no concentration change as purchased analytical grades with an assay of 99%. Under bias voltage of ±1 V to Y-OF, illumination of light (*λ*_365 nm_) at an intensity of 24 mW/cm^2^ (UV-ON) exhibits an ohmic behavior at Au/ZnO/Au interfaces with an increase in dark current (*I*_D_~40 nA) to the photocurrent (*I*_ph_) of ~18.36 μA (Fig. 2a)^22,23^. Further, Fig. 2a includes the VOC-dependent *I*–*V* curves (UV-T, UV-B, UV-A, UV-E, UV-D, and UV-M) demonstrating the substantial changes in the interfacial conduction path due to the VOC parameters^24^. The photocurrent reduces with triethylamine, benzene, acetone, ethanol, and methanol (~0.76, 0.90, 2.05, 4.09 , and 13.12 μA), whereas decanol interaction produces the photocurrent response of ~19.15 μA having less significant variation from the UV-ON condition (*I*_ph_~18.36 μA). Fig. 2b illustrates the sensitivity of Y-OF as a function of VOC represented with changes in the photocurrent response, Δ*I*_ph_ = *I*_ph (VOC)_–*I*_ph_. The suitability of VOC to serve as logic input sources (I/P) is optimized based on the photocurrent factor, where decanol with a high response is fixed for binary ‘1’ and other solvents with a reduced response occupy the ‘0’ value (Fig. 2c). A simple schematic view of Y-OF operating as the logic gate (OR and AND) is illustrated in Fig. 2d. Figure 2e–g shows the pictorial images of the designed experimental setup to investigate the piezophototronic effect based on optofluidic gate switching.

### Demonstration of the OR gate

**Fig. 3:**
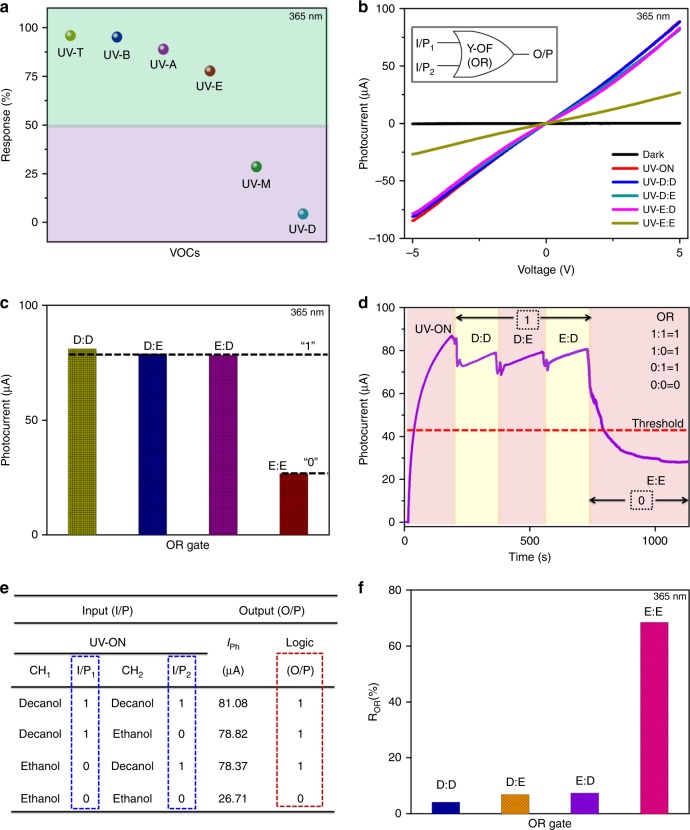
Demonstration of the optofluidic OR logic gate. **a** Response rate of Y-OF under VOC I/P. **b** *I*–*V* curve obtained when operated with ethanol (E) and decanol (D) as binary operands. **c** Comparison of ON (‘1’) and OFF (‘0’) states with photocurrent switching. **d** Time-responsive analysis (*I*–*t* graph) of Y-OF functioning of the OR operation. **e** Experimental truth table of the measured input (I/P_1_, I/P_2_) and output (O/P) conditions. **f** Corresponding Y-OF sensitivity as the OR gate

To demonstrate the OR gate using the as-fabricated Y-OF device, two fluids for binary conditions are opted based on the sensitivity differences between the VOC determined in terms of its response, $R\left( \% \right) = \frac{{R_{\left( {{\mathrm{UV}} - {\mathrm{VOC}}} \right)} - R_{\left( {{\mathrm{UV}} - {\mathrm{ON}}} \right)}}}{{R_{\left( {{\mathrm{UV}} - {\mathrm{ON}}} \right)}}} \times 100$, where *R*_(UV–ON)_ is the photocurrent response in the absence of fluids and *R*_(UV–VOC)_ is the photocurrent response in the presence of fluids^25^. Fig. 3a shows the calculated response for different VOC interactions to ZnO NR. Decanol having the least response is chosen for input source ‘1’ and ethanol with a notable difference in its responsivity with that of decanol is fixed for ‘0’ I/P source. The photoresponsivity (*R*_λ_) under the UV fluid is improved to 1.11 mA/W (UV ethanol) and 3.38 mA/W (UV decanol) as the bias voltage is increased from ± 1 to ± 5 V (Supplementary Fig. 5). Fig. 3b presents the *I*–*V* graph of the OR gate operation under a bias voltage of ± 5 V with coupled effects of light and fluidic sources. VOCs such as decanol (D) and ethanol (E) are passed through the inlet channels of Y-OF (CH_1_, CH_2_) under 365-nm illumination. During decanol in both channels (CH_1_ = D, CH_2_ = D) corresponding to the logic operands I/P_1_ = 1, I/P_2_ = 1, the *I*–*V* plot is observed with a high photocurrent of ~81.08 μA which maintains close to UV-ON. In binary terms, UV-D:D refers to the condition “1:1 = 1” in the OR gate. When the channels are operated with a nonidentical VOC (CH_1_ = D, CH_2_ = E) such that I/P_1_ = 1, I/P_2_ = 0, the photocurrent is maintained high with less deviation (~78.82 μA). The channels are interchanged with the fluid operands (CH_1_ = E, CH_2_ = D) such that I/P_1_ = 0, I/P_2_ = 1; Y-OF sustains with a high photocurrent (~78.37 μA). Therefore, UV-D:E and UV-E:D logically determines the OR functionalities at “1:0 = 1” and “0:1 = 1”. At both the channels with ethanol as the fluid source (CH_1_ = E, CH_2_ = E) referring to I/P_1_ = 0, I/P_2_ = 0, the photocurrent declines reaching a value of ~26.71 μA. A low photocurrent response ensures the condition “0:0 = 0” of the OR gate. The comparison of the Y-OF photocurrent under different VOC operands is shown in Fig. 3c.

More defined OR Boolean operations are demonstrated through time-dependent analysis (*I*–*t*) performed under a bias voltage of + 5 V. The UV-ON condition is maintained for a certain time (~190 s) until a constant photocurrent is observed and then fluidic conditions are performed. When the UV-D:D state is activated, the photocurrent steadily increases. Similarly at UV-D:E and UV-E:D states, high photocurrents are produced corresponding to ‘1’ logic output (O/P). When the UV-E:E state is ON, the photocurrent gradually decreases lower than the fixed threshold limit of <50% of the maximum photocurrent generated from the Y-OF device representing the ‘0’ logic output. Each state is maintained for a period of ~200 s to analyze evident variations in the switching behavior. The small drop observed between each switching condition is expected due to the disturbances experienced by the device while controlling the transfusion set roller to pass the fluids into the channels, and interchanging the channels based on input conditions. The truth table in Fig. 3e summarizes the modulations in the OR operand parameters and its corresponding sensitivity (*R*_OR_%) is presented in Fig. 3f. The results ensure the ideal functioning of the Y-OF device as the Boolean operator OR gate based on optofluidic tuning using light–chemical sensing mechanisms.

### Strain-induced intrinsic reconfiguration to logic AND

**Fig. 4:**
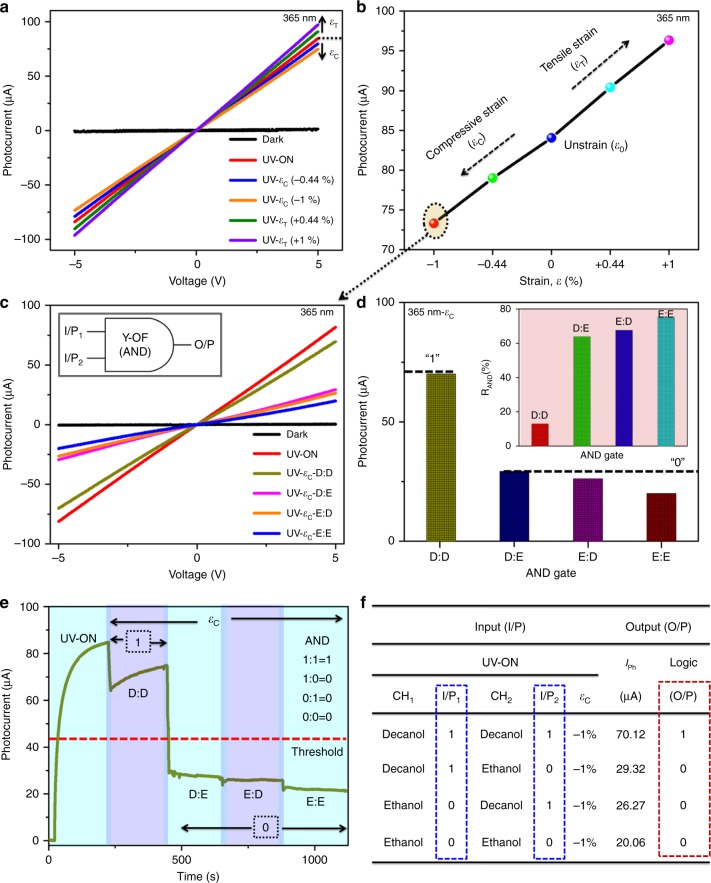
Piezophototronic triggered switching of Y-OF as the AND gate. **a** Dependent photocurrent response based on compressive (*ε*_c_)/tensile (*ε*_T_) strain conditions. **b** Corresponding comparison plot of strain vs. photocurrent. **c** An active AND gate with optical–fluidic–strain inputs and its electrical output (*I*–*V*). **d** Sensitivity of AND logic. **e** Piezophototronic configured AND gate as a function of time (*I*–*t* graph). **f** Experimental truth table of the operating AND conditions determining high (‘1’) and low (‘0’) states

To meet the present demands in the implementation of multiple logic devices with the same analyte (fluids), we demonstrated the fabricated Y-OF to perform the AND functionality with no further changes in the optical and fluidic parameters enabled through integration of the piezophototronic concept^26,27^. We first determined the suitable strain (*ε*) conditions to switch the OR gate to the AND gate directly. Fig. 4a shows the *I*–*V* graph with changes in the photocurrent when the device is strained (compressive/tensile). As observed, the exertion of compressive strain (*ε*_c_) on the device decreased the photoresponse due to the generation of negative piezoelectric potentials (*σ*^−^) from PVDF/ZnO NR. The piezocharges get accumulated at the MSM interfaces by which the barrier height increases. In the presence of tensile strain (*ε*_T_), the photocurrent increases due to the positive piezoelectric potentials (*σ*^+^) aligned at the MSM interfaces which reduced the barrier heights^26^. The interfacial gating mechanism of strain-induced behavior is illustrated schematically in Supplementary Fig. 7.

The strain % acting on the device is estimated to be + 0.44%, +1% (tensile condition), and −0.44%, −1% (compressive condition) calculated using $\varepsilon _{{\mathrm{C/T}}} = 3\frac{a}{l}\frac{{D_{{\mathrm{max}}}}}{l}\left( {1 - Z/l} \right)$ where *a* and *l* are the thickness and length of substrates, *D*_max_ is the maximum bending distance of the device using the rod, and *Z* is the actual distance moved by the rod (the distance moved by the rod is read using the clamp scale (Fig. 2g))^23^. Fig. 4b illustrates the deviation of photocurrent values at different strain conditions. To perform the AND gate, the compressive strain of −1% is preferred as a suitable strain rate as it exhibits the maximum reducing nature in the current response, and hence the lowered photocurrent value is utilized to switch the gate functions.

Fig. 4c shows the *I*–*V* plot of −1% strain exhibiting the AND operation in Y-OF. In the UV-*ε*_c_-D:D condition, the photocurrent produced is high (~70.12 μA) corresponding to “1:1 = 1” operation of the AND gate. As an influence of −1% strain, the photocurrent shows a reduced value compared with the unstrain condition, however, falling above the threshold limit accounting to the AND output logic of ‘1’ (Supplementary Fig. 8). By performing the UV-*ε*_c_-D:E condition that is I/P_1_ = 1, I/P_2_ = 0 under −1% strain, the photocurrent drastically drops to ~29.32 μA. A related effect is observed with a reduced photocurrent of ~26.27 μA when the channels are reversed for the UV-*ε*_c_-E:D condition such that I/P_1_ = 0, I/P_2_ = 1. The lowered photocurrent values by generating the negative piezoelectric potentials enabled the switching of logic values to ‘0’ corresponding to the binary AND modes of “1:0 = 0” and “0:1 = 0”. Under UV-*ε*_c_-E:E, the photocurrent response further reduces to ~20.06 μA that agreed with the Boolean condition “0:0 = 0” in the AND gate^28^.

The photocurrent and its corresponding response factor (*R*_AND_%) under −1% strain are illustrated in Fig. 4d. Real-time AND gate performance was demonstrated through time-dependent switching analysis (*I*–*t*) as shown in Fig. 4e. As observed in *I*–*V* behavior, the photoresponse gradually increases in the UV-*ε*_c_-D:D state relating to ‘1’ logic output (high). By maintaining the strain rate, we tested the Boolean operations corresponding to the input factors UV-*ε*_c_-E:D, UV-*ε*_c_-D:E, and UV-*ε*_c_-E:E. It shows a reduced response that falls below the threshold limit which are read as ‘0’ logic outputs (low). The results are summarized in the truth table of Fig. 4f with a specific AND gate operation using Y-OF. The systematic analysis illustrates the behavior of the piezophototronic effect to intrinsically reconfigure OR to the AND gate by compiled means of light–fluid–strain-induced mechanisms.

Further, the reliability of the device performance is evaluated through repeatability studies (Supplementary Figs. 10 and 11). The irradiated 365 nm UV source penetrates through the fluidic channel without any hindrance of light absorption by ethanol and decanol (Supplementary Figs. 12 and 13). Post the experimental studies, we analyzed the PVDF/ZnO NR film in Y-OF through FESEM (Supplementary Fig. 14) which confirms that there is neither detachment of the nanorods from the flexible PVDF substrate nor deformation in the morphological structure of ZnO NR. This assures the stability and reusability of the device. The preference of ethanol over the other ‘0’ state VOC listed in Fig. 2c is illustrated in Supplementary Fig. 15. Besides, to validate the generation of piezoelectric potentials, we carried out piezoelectrical analysis by using an electrometer (Supplementary Fig. 18). The Y-OF device delivered a peak-to-peak piezoelectric voltage (*V*_oc_) of ~0.7 V and current (*I*_sc_) of ~10 nA through mechanical stimuli of 2 N force (F) (Supplementary Fig. 19).

### Mechanism of piezophototronic gated optofluidic logic switching

**Fig. 5:**
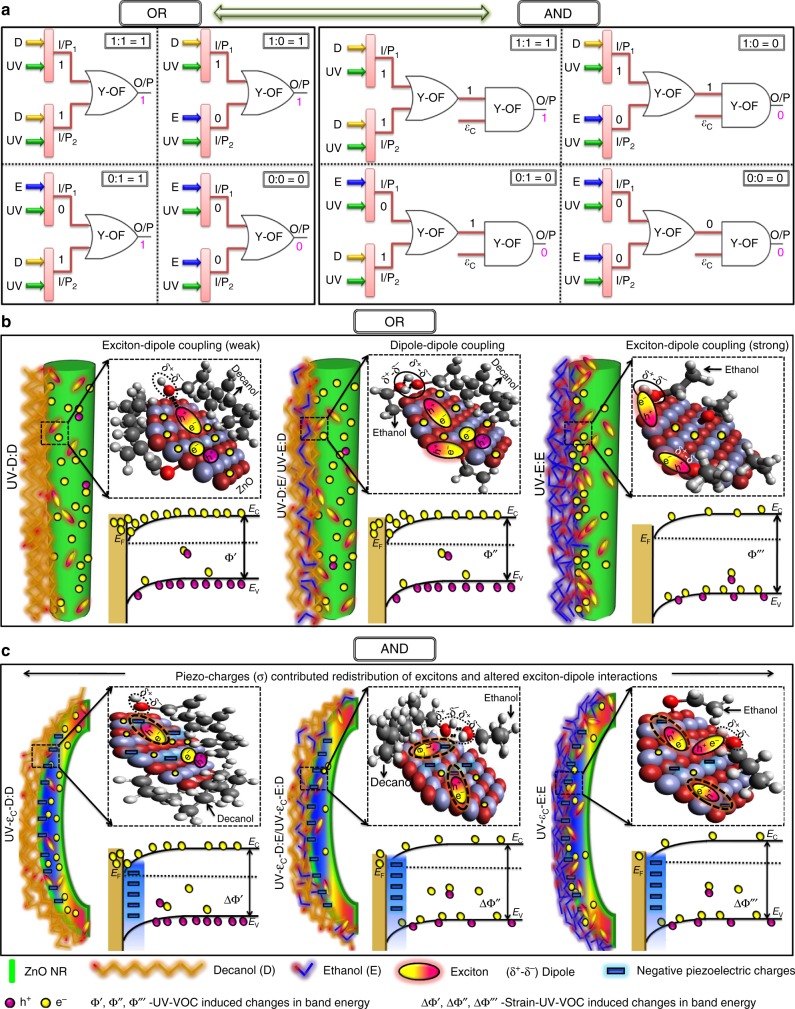
Mechanism of piezophototronic-based optofluidic logic gates (PPOF). **a** Logic circuitry representation of Y-OF-based hybrid switching configuration of OR↔AND Boolean operators. **b** Interactive scheme of ethanol (E) and decanol (D) with ZnO NR activating the OR logic states with bandgap illustrations UV-D:D, UV-D:E/UV-E:D, and UV-E:E. **c** Interactive scheme of ethanol (E) and decanol (D) with ZnO NR reconfigured to the AND gate with negative piezocharge-tuned band alignments UV-*ε*_c_-D:D, UV-*ε*_c_-D:E/UV-*ε*_c_-E:D, and UV-*ε*_c_-E:E

The working of the piezophototronic gated optofluidic switching of logic devices involving the coupled effects of piezoelectric (*ε*_c_)-optical (*λ*_365 nm_)-chemical sensing (VOC) is illustrated schematically in Fig. 5. Fig. 5a demonstrates the logic diagram of the optofluidic-controlled OR gate and the corresponding piezophototronic-enabled reconfiguration to the AND Boolean operator. The experimental sensing mechanism governing the OR logic scheme is illustrated in Fig. 5b through the Y-OF parametric configurations involving VOC–ZnO NR interactions. At room temperature, ZnO NR exposed in the dark absorbs the O_2_ molecules $({\mathrm{O}}_2 + {\mathrm{e}}^ - \to {\mathrm{O}}_{2\;({\mathrm{abs}})}^ - )$ from the atmospheric environment. The ${\mathrm{O}}_{2\;{\mathrm{(abs)}}}^ -$ traps the electrons in the conduction band and reduces the free charge carriers, which hinders the sensitivity of ZnO NR to fluidic interactions. The chemisorbed ${\mathrm{O}}_{2\;({\mathrm{abs}})}^ -$ ions are thermally highly stable, which are difficult to remove at room temperature, thus making the device less responsive when exposed to fluids. Under the UV source, the photogenerated electrons and holes $(hv \to {\mathrm{e}}^ - + {\mathrm{h}}^ + )$ cause photoabsorption of the chemisorbed ${\mathrm{O}}_{2\;({\mathrm{abs}})}^ -$ and release the trapped electrons to move freely$({\mathrm{O}}_{2\;({\mathrm{abs}})}^ - + {\mathrm{h}}^ + \to {\mathrm{O}}_2)$ producing an increased current response^29^. With no hindrance of ${\mathrm{O}}_{2\;({\mathrm{abs}})}^ -$, the optical source promotes better interactions of VOC–ZnO NR in order to explore the optofluidic-based switching behaviors. With the improved VOC–ZnO NR effect, we examine the picture of systematic tuning using the ethanol and decanol as fluidic sources.

The VOC molecules produce a net dipole moment which controls the surface charges and interfacial barriers (molecular gating), thereby enabling chemical sensing characteristics^30–34^. Fig. 5b illustrates the coupling at the UV-D:D condition. With decanol, the photocurrent is high due to low diffusivity of decanol over the ZnO NR surface. With lengthier hydrocarbon chains, decanol is hard to form dissociated ions (${\mathrm{OH}}^ - /{\mathrm{H}}^ +$) trapping the photogenerated charge carriers. Further, the dipole moment (1.61 D) of decanol facilitates weak exciton–dipole interaction and so the ionic transfer resistance does not dominate the electrical conduction path of MSM interfaces^35–37^. With increased chain length and the less pronounced dipole effect between decanol and ZnO NR, the photocurrent at UV-D:D is high due to reduced positive charges which had altered the surface charge distribution of ZnO NR. In the case of UV-E:D and UV-D:E states (Fig. 5b), where ethanol and decanol meet at the converging point of the Y-channel, the heterogeneous liquid phase is formed with the fusion of two different VOCs. The mixture of dual solvent is expected to exist in the form where long-chained decanol with higher density strongly encapsulates ethanol with cross-linked intermolecular dipoles (dipole–dipole coupling). The effectiveness of the dipole moment decreases with the cross-linking effect which causes fewer chances of the exciton–dipole interaction between ZnO NR and ethanol^38^. Therefore, we allow effective conduction of photogenerated charge carriers at the MSM interfaces. In the UV-E:E condition (Fig. 5b), with only the presence of ethanol $\left( {{\mathrm{C}}_2{\mathrm{H}}_5{\mathrm{OH}} + 3{\mathrm{O}}_{2\;(hv)}^ - \to 2{\mathrm{CO}}_2 + 3{\mathrm{H}}_2{\mathrm{O}} + 3{\mathrm{e}}^ - } \right)$ creates a strong exciton–dipole interaction between Zn and O to molecular dipoles in ethanol. Ethanol with a lone pair of electrons tends to couple effectively with the excitons facilitating the changes in the ZnO surface charge distributions, thereby consequently increasing the MSM band bending. This shifts the photocurrent toward a lower value as an indication of interacted molecular dipoles and reduces negative surface charges as a result of surface-state effects^33,38^. Besides, the chemical sensing depends on the concentration of ethanol (99%). Unlike a low concentration of ethanol which contributes charged species through two-step chemisorption-dissociated ions $\left( {{\mathrm{H}}_2{\mathrm{O}} + {\mathrm{Zn}} + {\mathrm{O}}_{\mathrm{o}} \! \leftrightarrow \! 2{\mathrm{OH}} - {\mathrm{Zn}} + {\mathrm{V}}_{\mathrm{o}} + 2{\mathrm{e}}^ - ;{\mathrm{H}}^ + + {\mathrm{O}}^{2 - } \! \leftrightarrow \! {\mathrm{OH}}^ - } \right),$ the higher concentrated ethanol having less water molecules hinders the donation of charge carriers. Also, the liquid ethanol forms an effective shielding layer over the ZnO NR^39^. The effective shielding centers decrease the diffusion of OH^−^ ions based on the ion diffusion-limited mechanism and it also buckles the photogenerated electrons in the conduction band. Hence, a lowered photocurrent using ethanol is observed as a systematic contribution from exciton–dipole coupling (dipole moment, 1.68 D) and formation of shielding centers (concentration level).

The demonstrated intrinsic reconfiguration phenomena with the compressive strain (*ε*_c_) of −1% performing sequential logic of AND are illustrated in Fig. 5c. The photocurrent at the UV-D:D state is high due to the weak exciton–dipole interaction which also refers to the AND operation “1:1 = 1”. Meanwhile, the applied strain induces an internal ZnO lattice deformation which produces piezocharges (*σ*^+^/*σ*^−^) due to its nonsymmetric crystal arrangements^40^. The charge ( ± ) of piezopotentials depends on the nature of the applied strain (tensile/compressive), where *ε*_c_ produces negative piezoelectric potentials (*σ*^−^) at MSM interfaces^23,28^. In the UV-*ε*_c_-D:D condition, the negative piezocharges screen the excitons which in turn altered the exciton–dipole coupling. The shift in the photocurrent from UV-D:D (~79.42 μA) to UV-*ε*_c_-D:D (~70.12 μA) reveals the less significant effect of an altered exciton–dipole coupling in affecting the current flow (as decanol possesses a weak exciton–dipole impact on ZnO surface states). Therefore, the reduced photocurrent is expected due to the widening of space charge regions contributed by the accumulated piezocharges, which increases the barrier heights; however, with the photocurrent above the threshold limit, UV-*ε*_c_-D:D still accounts to logic “1:1 = 1”. When UV-*ε*_c_-D:E and UV-*ε*_c_-E:D conditions are enabled, the negative piezopotentials perform redistribution of the excitons which had improved the exciton–dipole coupling^33^. Also, with the increased band alignments which reduce the photonic charge transfer across the interfaces, the photocurrent produced is low by thus switching the output states to logic ‘0’. At the UV-*ε*_c_-E:E condition, the photocurrent drops further due to the synergetic effect of the piezopotential to the exciton interaction in enhancing the exciton–molecular chain interfaces and barrier effects^33^. The demonstrated cascaded optofluidic logic device with the piezophototronic switching concept by using Y-OF provides a versatile and significant mechanism to perform multiple logic functions with intelligent, adaptable interactions (optical–chemical–strain) in shaping the electronic and biomedical device properties^41^.

## Discussion

Our study on Y-OF presents a flexible framework to perform binary computations with an integrated piezophototronic mechanism controlling the optofluidic switching of logic gates (PPOF). The interaction between VOC, ethanol (E), and decanol (D) chemically stimulates the ZnO NR photonic charge carriers complementing the Boolean operands (I/P) ‘1’ and ‘0’. By tuning the input states, the measured photocurrent of Y-OF configures the OR operation. With the integration of piezoelectric potentials through compressive strain (*ε*_c_) of −1%, Y-OF reconfigures intrinsically to logic AND. Transfer of dual-logic switching in one design (Y-OF) with the integration of the optical–fluidic–strain concept benefits to organize complicated micro/nano electro-optofluidic systems in a chip. The intrinsic reconfiguration demonstrates a promising potential featuring the smart signal-responsive systems sophisticating the designing of futuristic (bio) molecular computers, binary-operating (chemical) biosensors, electrochemical transducers to switch biofuel cells, addressable logic memory units, modeling of on-chip optical tweezers, and actuations, thus giving rise to programmable logic-triggered therapeutic and diagnostic detection tools.

## Methods

### Synthesis of the sensing layer

Zinc oxide nanorods (ZnO NR) are grown on the PVDF film through the in situ method followed by hydrothermal process^22^. Ionization of the PVDF film is carried out with Zn^2+^ through the addition of 0.4 g of zinc acetate (Zn(CH_3_COO)_2_·2H_2_O, Alfa Aesar) into 4 g of polyvinylidene fluoride (PVDF, Alfa Aesar) dissolved in 5:3 volume% of N-methyl-2-pyrrolidone:acetone solvents. The solution is probe sonicated at an amplitude of 35% for 1 h and then polymerized at 70 °C overnight. Later, the ionized PVDF film is treated hydrothermally with an equal molecular ratio of Zn(CH_3_COO)_2_·2H_2_O and hexamethylenetetramine (HMT, Alfa Aesar) for 12 h at 80 °C. Finally, the PVDF–ZnO NR film is washed with ethanol and water and dried at 70 °C.

### Fabrication of Y-OF

Y-OF of the metal–semiconductor–metal (MSM) type is designed by using the PVDF–ZnO NR film attached onto a polyethylene terephthalate (PET) sheet. The MSM interfaces are developed using a gold (Au) electrode sputter deposited (using Quorum Q300T D system with high-quality vacuum, 5 × 10^−3^–5 × 10^−2^ mbar at 20 mA of deposition current) on top of the ZnO NR with a spacing of ~1 mm. Two copper (Cu) wires are attached on each Au electrode using silver (Ag) paste. Later, the Y-shaped channel is formulated by using polydimethylsiloxane (PDMS, Dow Corning) ensuring a pathway of two inlet channels (CH_1_/CH_2_) converging to form an outlet flow of fluids. The Y-shaped PDMS groove is carefully attached on top of PVDF–ZnO NR/Au in a manner that the channel exactly lays in the 1 mm spacing area between the Au electrodes. This facilitates the fluids passing through the channels to have direct interaction with ZnO NR. Finally, the device is packed with another PDMS thin layer beneath the PET sheet, thus completing the fabrication of the Y-OF sensor. The dimension of Y-OF is 3 × 2 cm, which includes 1 × 1 cm PVDF–ZnO NR film.

### Experimental setup

We developed a structure to smooth the path of experimental approaches toward piezophototronic-tuned optofluidic logic gates (Fig. 2e–g). It constitutes four syringes (S_1_, S_2_, S_3_, and S_4_) with a transfusion set (TS) attached to each syringe. The roller of the TS is used to control the flow of fluids into the channels of Y-OF. The outlet end of the Y-OF device is placed firmly onto a stand attached to the base of the clamp. The inlet end of Y-OF is allowed to move freely where the TS needles are inserted into the channels (CH_1_/CH_2_). Flexible tubes are used as a support to connect the needles with the channels so that the Y-OF device can bend without restriction. A rod (R) is fixed on the clamp to move horizontally by which the free end of the device bends experiencing an external mechanical strain (*ε*). Further, an optical fiber from a LED source meter is fixed near the device area. The experiment is carried out in the presence of a black box which excludes the environmental light influencing the performance of Y-OF.

### Instrument specifications

Photocurrent measurements were performed using the CV-IV parameter analyzer, Agilent-B1500A measured from 1 pA/0.5 V to 100 mA/100 V of semiconductor devices in variable frequency ranges (1 kHz–5 MHz). The LED meter operating at a wavelength (*λ*) of 365 nm with variable light intensities was used as a UV source illuminator to test the Y-OF characteristics. The piezoelectric performances were analyzed by using a high-impedance electrometer (Keithley 6514). To estimate the penetration depth of light in the fluids of the UV-Vis spectrophotometer Lambda25, PerkinElmer was employed, respectively.

## Supplementary information

Supplementary Information

Description of Additional Supplementary Files

Supplementary Video 1

## Data Availability

The authors declare that the data supporting the findings of this study are available within the article and its Supplementary Information files. All the other data supporting the findings of this study within the article are available from the corresponding author upon reasonable request.
