# Supplementary material for: Piezophototronic gated optofluidic logic computations empowering intrinsic reconfigurable switches
Source: Nat Commun. 2019 Sep 26;10:4381. doi: 10.1038/s41467-019-12148-y (PMC6763476; doi:10.1038/s41467-019-12148-y)
Supplement: Supplementary file 1 — Supplementary Information [file 41467_2019_12148_MOESM1_ESM.docx]

**Supplementary Information**

**Piezophototronic Gated Optofluidic Logic Computations Empowering Intrinsic Reconfigurable Switches**

*Yuvasree Purusothaman^†^, Nagamalleswara Rao Alluri^†^,* [*Arunkumar Chandrasekhar*](http://pubs.acs.org/author/Chandrasekhar%2C+Arunkumar)*^††^, Vivekananthan Venkateswaran^†^,* [*Sang-Jae Kim*](http://pubs.acs.org/author/Kim%2C+Sang-Jae)[*^*^*](http://pubs.acs.org/doi/abs/10.1021/acsami.6b00548#cor1)*^†^*

*^†^ Nanomaterials and System Lab, Department of Mechatronics Engineering,*

*Jeju National University, Jeju 690756, Republic of Korea*

Department of Sensor and Biomedical Technology

School of Electronics Engineering

Vellore Institute of Technology

Vellore 632014, India

*^††^Department of Sensor and Biomedical Technology, School of Electronics Engineering,*

*Vellore Institute of Technology, Vellore 632014, India*

*^*^Corresponding Author*

*Prof. Sang-Jae Kim (S-J Kim),*

*Fax: +82-64-756-3886; Tel: +82-64-754-3715,*

*Email ID:kimsangj@jejunu.ac.kr*

**
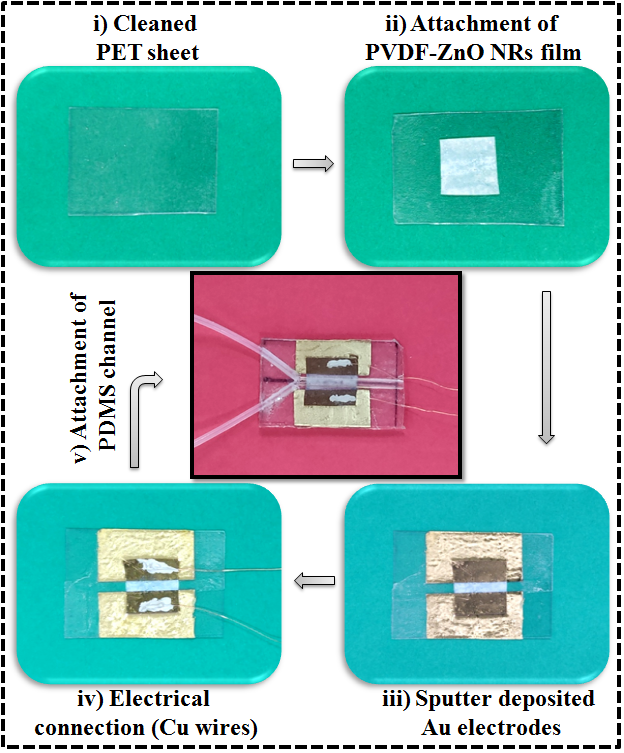
**

**Supplementary Figure 1** Optical images of Y-OF device fabrication.


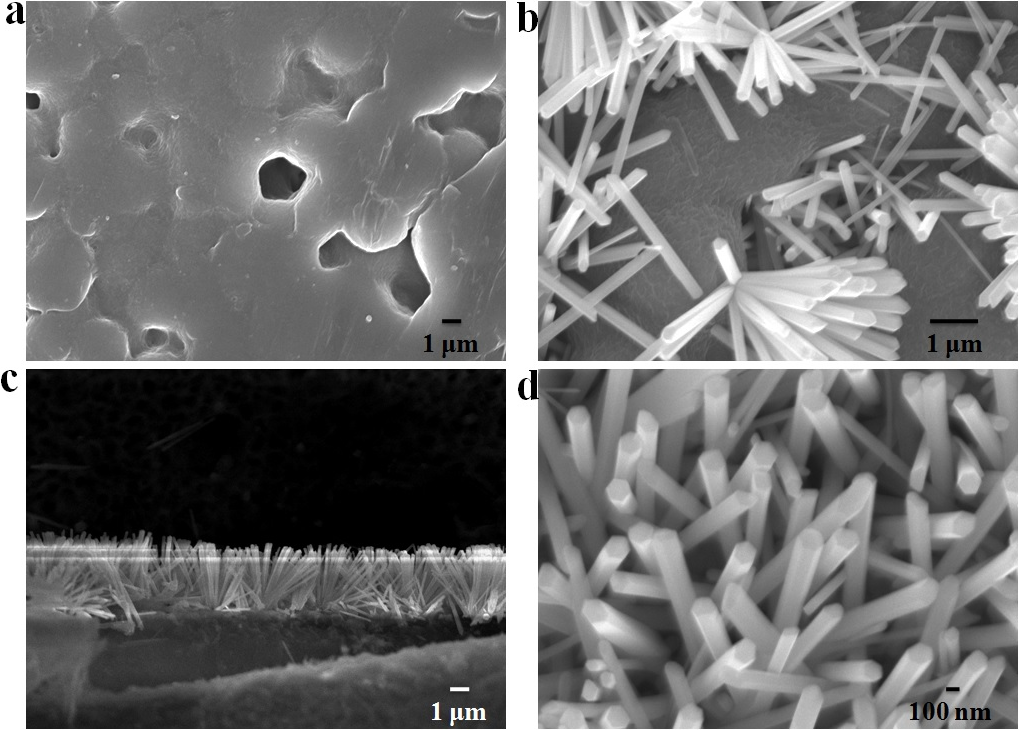


**Supplementary Figure 2** FESEM images of PVDF/ZnO NRs film. **a** Ionized PVDF film before the hydrothermal process. **b** Grown ZnO NR in ionized PVDF (after the hydrothermal treatment). **c** Cross-sectional view of film illustrating well-ordered growth of ZnO NR on PVDF. **d** Magnified surface view of ZnO NR.


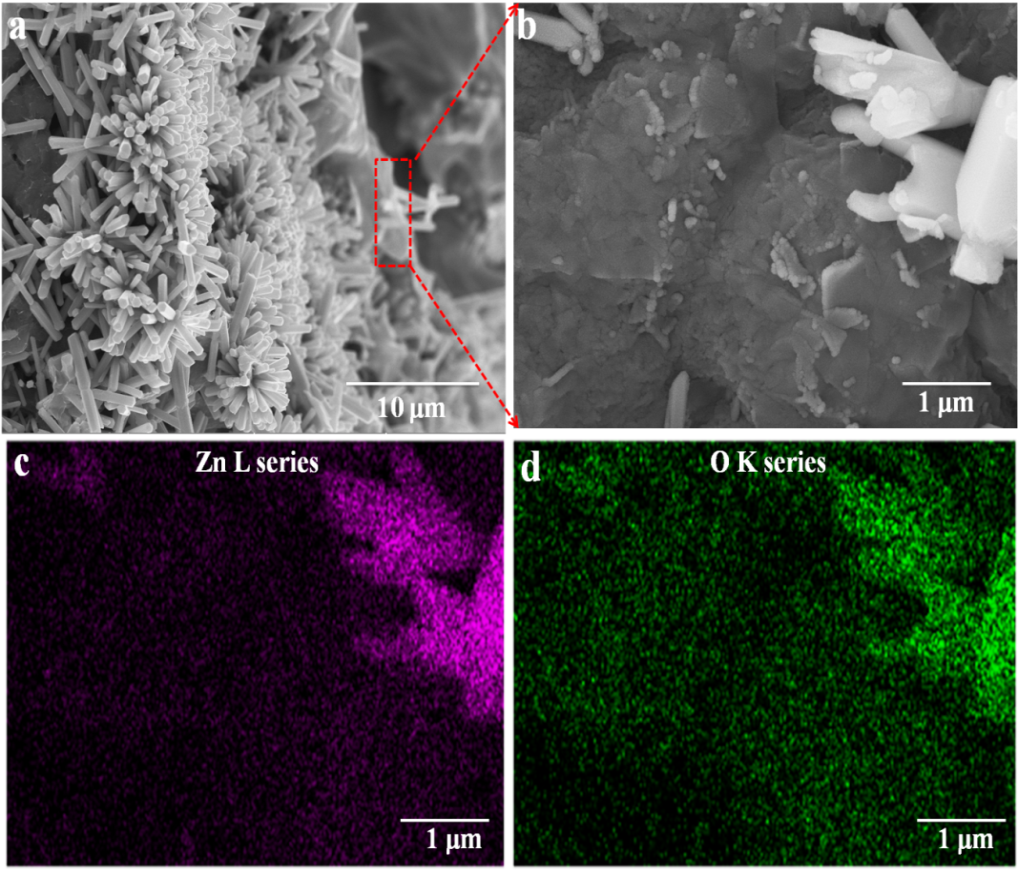


**Supplementary Figure 3** **a, b** FESEM images of ZnO NR/PVDF substrate. **c, d** EDS mapping of Zn and O species.


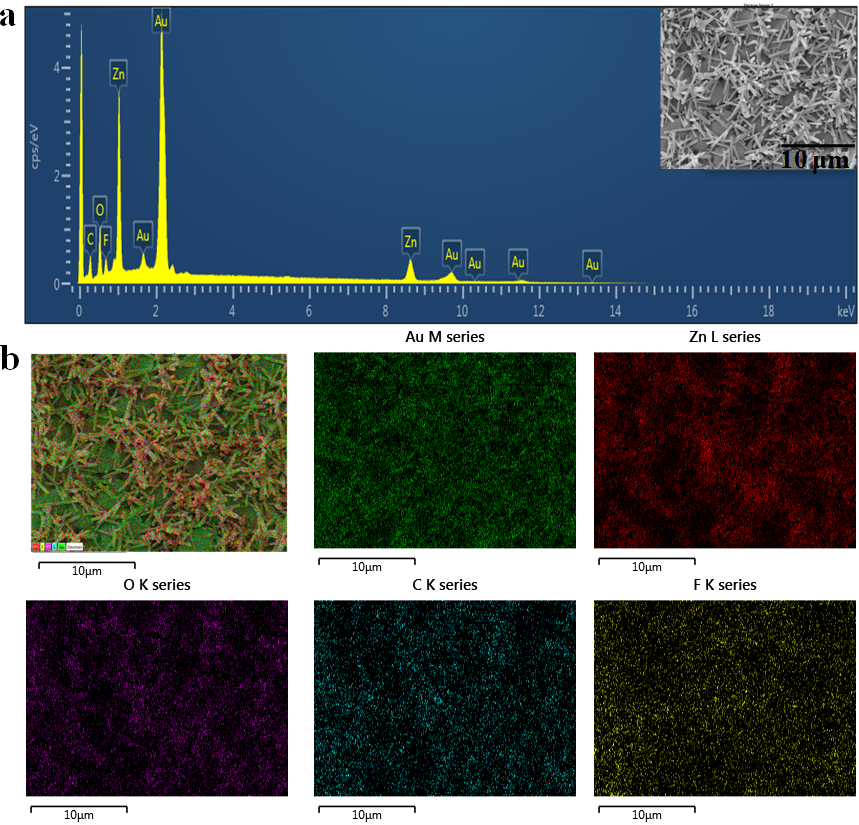


**Supplementary Figure 4** **a** Elemental analysis of Au-PVDF/ZnO NR film. **b** Corresponding EDS mapping with the panels of Au, Zn, O, C, F (C and F mapped from the PVDF substrate).

**Bias dependent photo responsivity:**

The bias improves the photocurrent^1^ (UV-ON condition) from 18.36 μA to 85 μA as the voltage increases from ± 1 V to ± 5 V. This enhances the photo responsivity^2^ ($R_{\lambda})$ of Y-OF device from 0.76 mA/W (± 1 V) to 3.54 mA/W (± 5 V), and so improves the signal strength of Y-OF sensing when operated further under fluid parameters. Y-OF with UV-fluid condition shows enhanced photo responsivity of 0.17 mA/W to 1.11 mA/W (UV-ethanol) and 0.80 mA/W to 3.38 mA/W (UV-decanol) as the bias voltage is changed from ± 1 V to ± 5 V (supplementary Figure 5).

$R_{\lambda}=\frac{I_{Ph}-I_{D}}{P_{L}\times S}$----------------- (1)

where, $I_{Ph}$ is the UV induced photocurrent, $I_{D}$ is the dark current response, $P_{L}$ is the incident power intensity (24 mW/cm^2^), and $S$ is the active area of the device (1 cm $\times$ 1 cm).


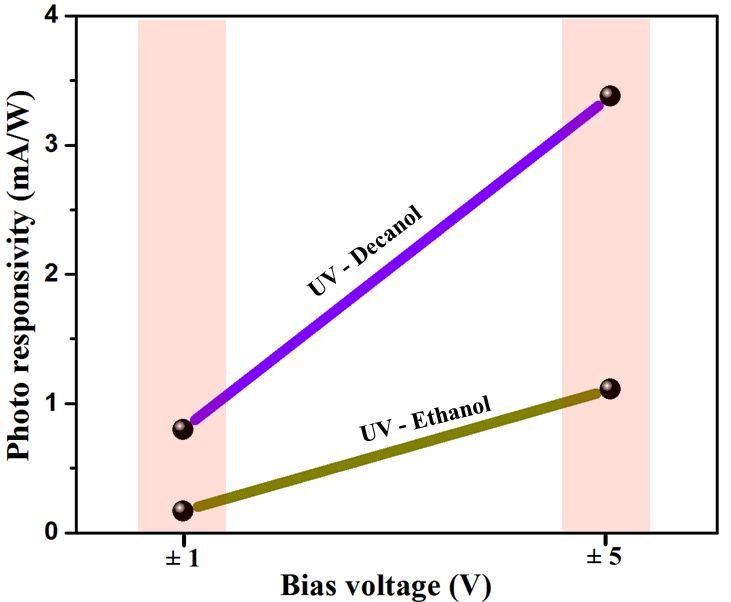


**Supplementary Figure 5** Bias dependent photo responsivity of Y-OF devices.

**Rise and decay time:**

The time responsive parameters ($\tau_{R}, \tau_{D}$) are determined from the exponential rise of photocurrent ($I_{Ph}$ to 63 % ($\sim1-e^{-1}$) of maximum $I_{Ph}$ and its exponential decay to 37 % ($\sim e^{-1})$ from maximum $I_{Ph}$^2^. Supplementary Figure 6 illustrates the current-time (I-t) curve under UV ON/OFF conditions with a rise time ($\tau_{r})$ of ~ 21 s and decay time ($\tau_{d})$ of ~ 50 s.


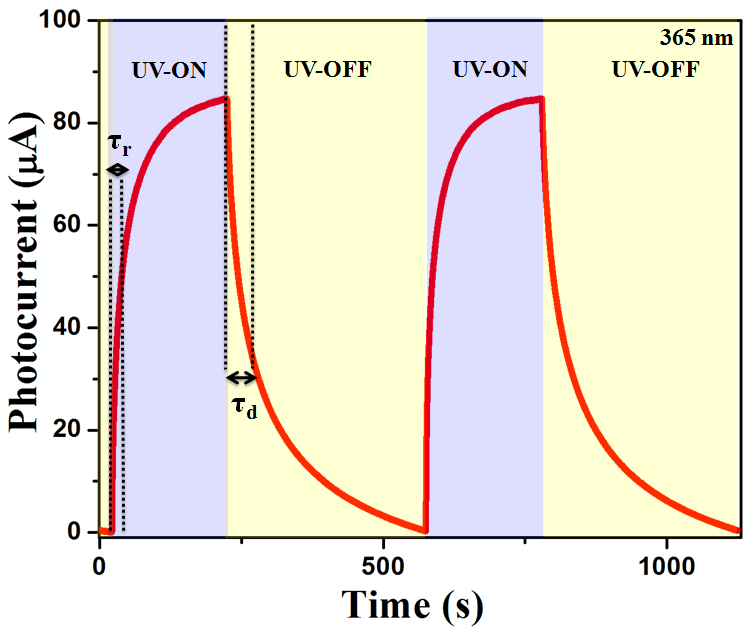


**Supplementary Figure 6** Time responsive analysis of Y-OF under UV (λ_365 nm_) ON/OFF conditions at irradiation intensity of 24 mW/cm^2^ (+ 5 V bias).

**Gating effects:**

The energy band diagram elaborates the interfacial gating of piezotronic and piezophototronic behaviors as a result of changes in the MSM barrier heights (Φ_1_/Φ_2_). Supplementary Figure 7a, b represents the band bending when there is no strain applied onto the Y-OF device. During tensile strain ($\varepsilon_{T}$, +1 %), positive piezoelectric polarization charges (σ^+^) are induced at both interfaces resulting in symmetric reductions in the Au-ZnO barrier heights. As a result, more photogenerated electronic transportations are enabled which improve the conductivity of Y-OF sensor thereby producing high current flow (supplementary Figure 7c, d). Under compressive strain ($\varepsilon_{C}$, −1 %), negative piezoelectric potentials (σ^−^) are generated, which raises the potential barriers at the metal-semiconductor interfaces that reduces the transportation of ZnO photo charge carriers (supplementary Figure 7e, f). The induced negative piezo-charges widen the space charge region at interfaces where the photogenerated carriers get neutralized due to the piezoelectric screening effect^3^. This influences the resistivity of ZnO NR to improve which reduces the photocurrent flow. Hence, the two interfaces are synchronously modulated in compressive direction by inducing negative piezoelectric charges under strain to switch from ‘1’ to ‘0’ state which reconfigures the logic of OR to AND gates.


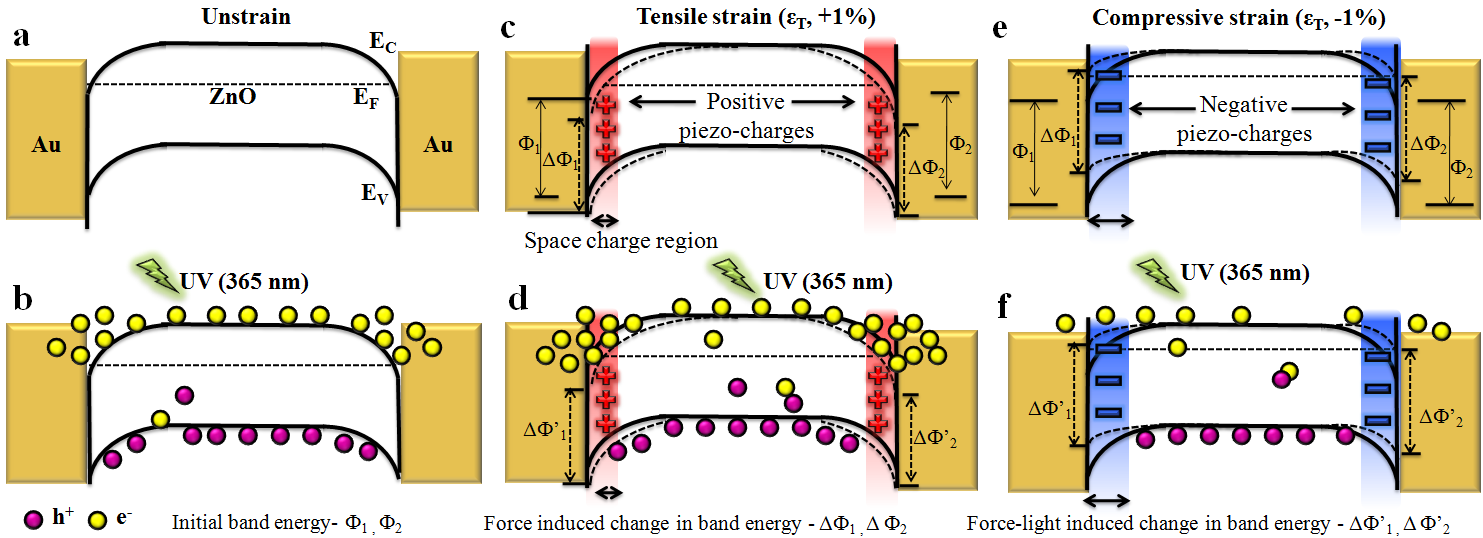


**Supplementary Figure 7** Energy bandgap diagram illustrating piezotronic/piezo-phototronic gating effects. **a, b** Band diagram of MSM interfaces without strain. **c, d** Tensile strain induced gating effect. **e, f** Compressive strain induced potential charge generation at interfaces.

**Effect of strain in UV-D:D state (UV-** $\boldsymbol{\varepsilon}_{\mathbf{C}}$**-D:D):**

Supplementary Figure 8a represents the I-V plot of Y-OF under the influence of −1 % strain in the presence of decanol in both the fluid inlets. As observed, the subjection of $\varepsilon_{C}$ reduces the photocurrent response to ~ 70.12 μA compared to unstrain 1:1 condition (~ 79.42); yet the reduced photocurrent falls above the threshold limit. In other words, the presence of $\varepsilon_{C}$ obviously causes changes in the photocurrent due to the piezophototronic effect; however, it makes no changes in computations of logic function ‘1’. For clarity of behavior, we represented the results compared to the operations corresponding to UV-D:D and UV- $\varepsilon_{C}$-D:D for AND logic in supplementary Figure 8b.

**
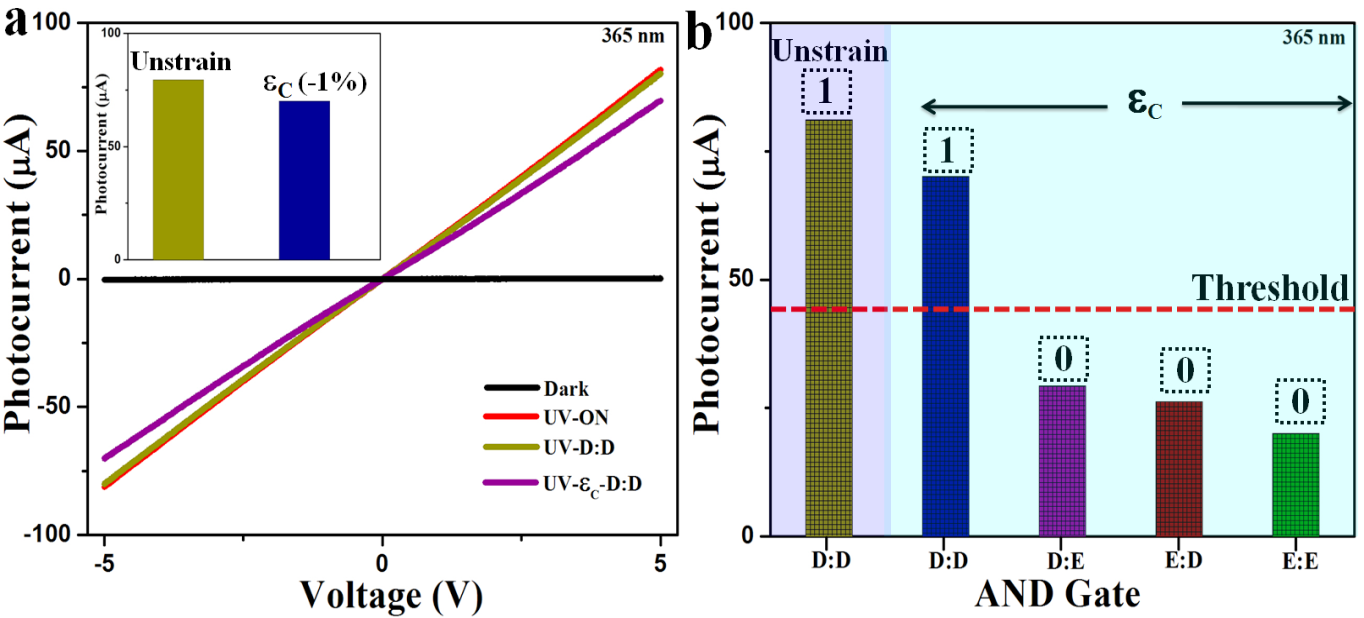
**

**Supplementary Figure 8** Effect of strain in AND gate for 1:1 (D:D) condition. **a** I-V response at $\varepsilon_{C}$ (−1 %). **b** Comparative plot of logic AND functionality with the influence of strain.

**I-V responses in log scale:**

Supplementary Figure 9a shows the log-log plots of VOC dependent photocurrent as a function of forward bias. The curve exhibits an increase in the photocurrent at higher voltage due to improvement in the ohmic behavior. The linearity of I-V characteristics at high biasing condition occurs from the space charge limited current conduction (SCLC) due to the increase in ZnO NR surface states with photo carriers distributed traps at the interfaces^4^. Supplementary Figure 9b shows the corresponding reverse bias semi-log plot of VOC dependent I-V response. Besides, the log scale of the I-V curve for the OR and AND gate performances are given in supplementary Figure 9c, d.


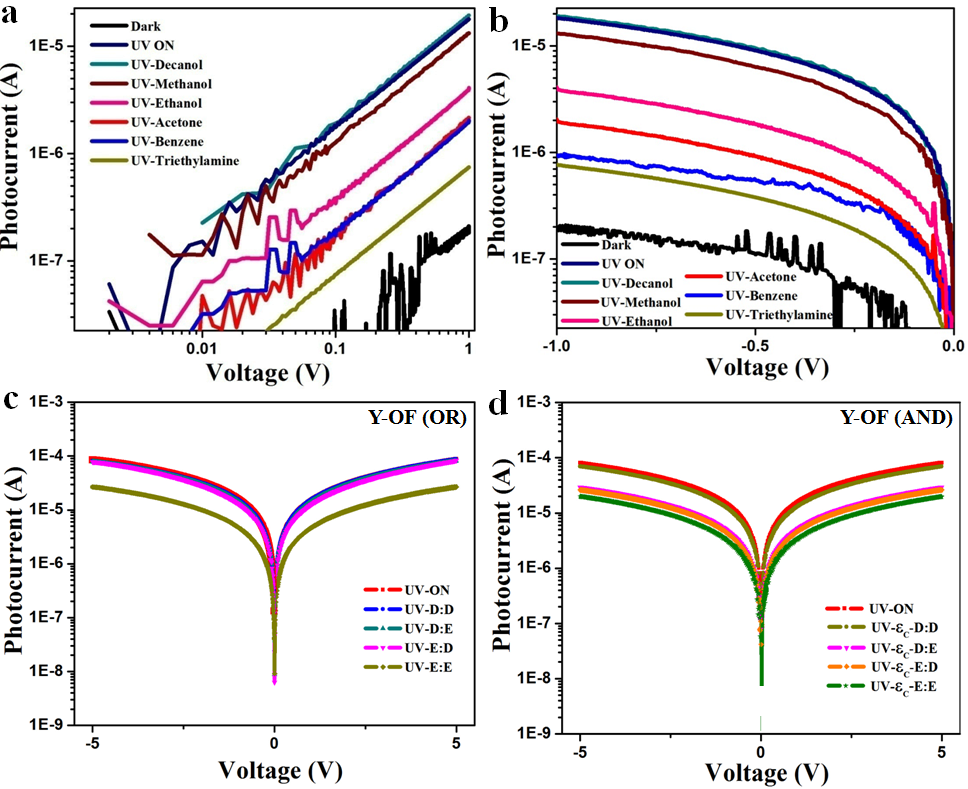


**Supplementary Figure 9 a** Log-log plot of VOC dependent I-V response (forward bias). **b** Semi-log plot of current (reverse bias) as a function of VOC. **c** Log scale of photocurrent at OR gate operation. **d** Log scale of AND gate operation.

**Repeatability and stability cycles:**

We fabricated additional Y-OF devices (D2, D3) to test the reproducibility of piezophototronic gated optofluidic logic switches. Supplementary Figure 10a, b represents the I-V analysis of OR and supplementary Figure 10d, e represents its intrinsic reconfiguration to AND gate. It ensures that Y-OF performs consistently producing ideal switching behavior with modulation of logic inputs as a function of fluid-strain-light sources. The comparative plot with error bar (supplementary Figure 10c, f) indicate the photocurrent variability of the sensor to be below $\pm$5 %. The deviation is expected due to the differences in carrier concentrations of ZnO NR with instability in the generation of vacancy sites/ point defects (Zn_i_, O_o_, V_o_)^5, 6^.

**
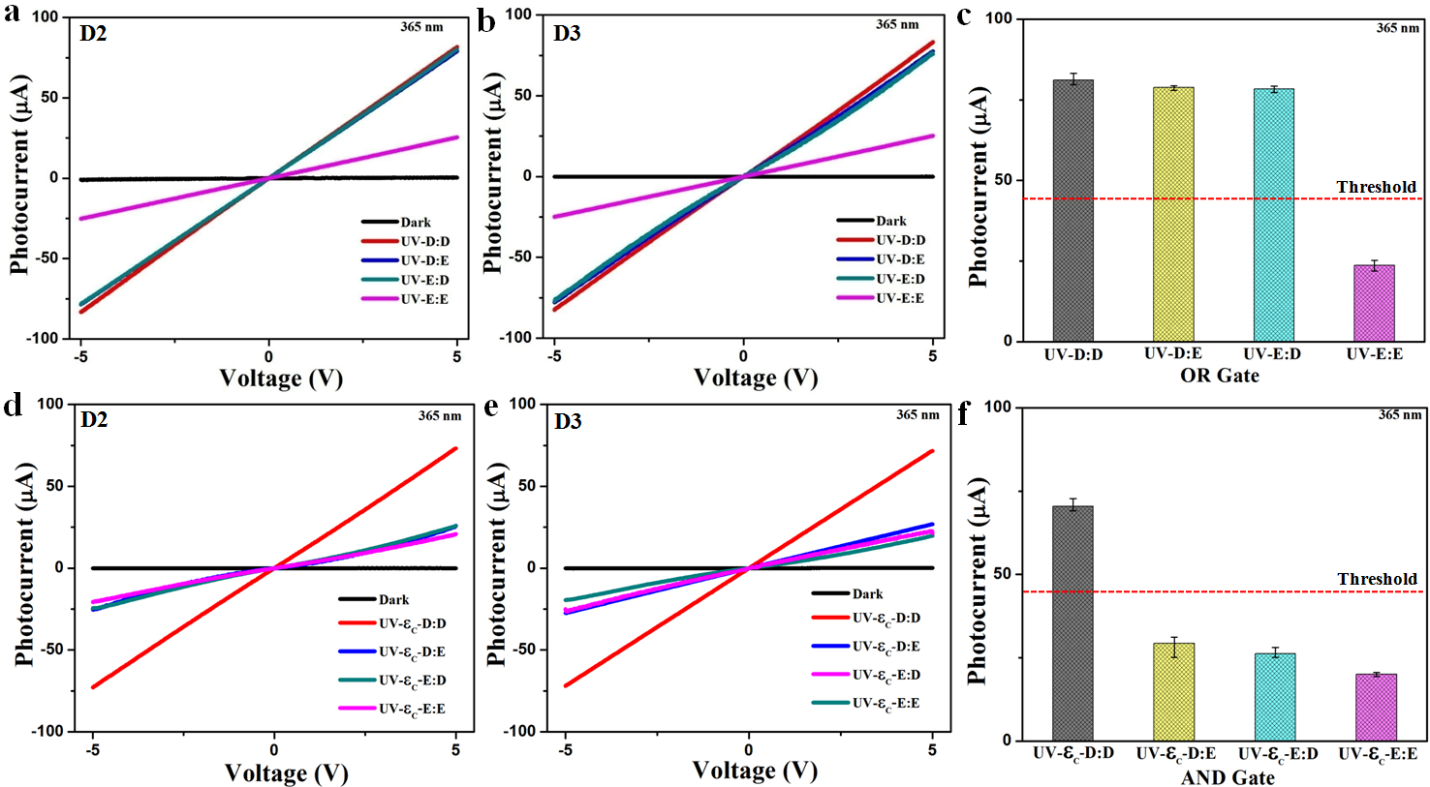
**

**Supplementary Figure 10** Reliability of Y-OF switching performance. **a, b** OR gate response. **c** Corresponding error bar. **d, e** AND gate switching with strain effect. **f** Error bar of AND operation.

The stability of the device is ensured through running for three cycles under OR-AND gate conditions. The results are shown in supplementary Figure 11a for OR gate and supplementary Figure 11b for AND gate with its corresponding I-V analysis. As observed from the plot, the device (D1) is capable to operate successfully for multiple cycles.

**
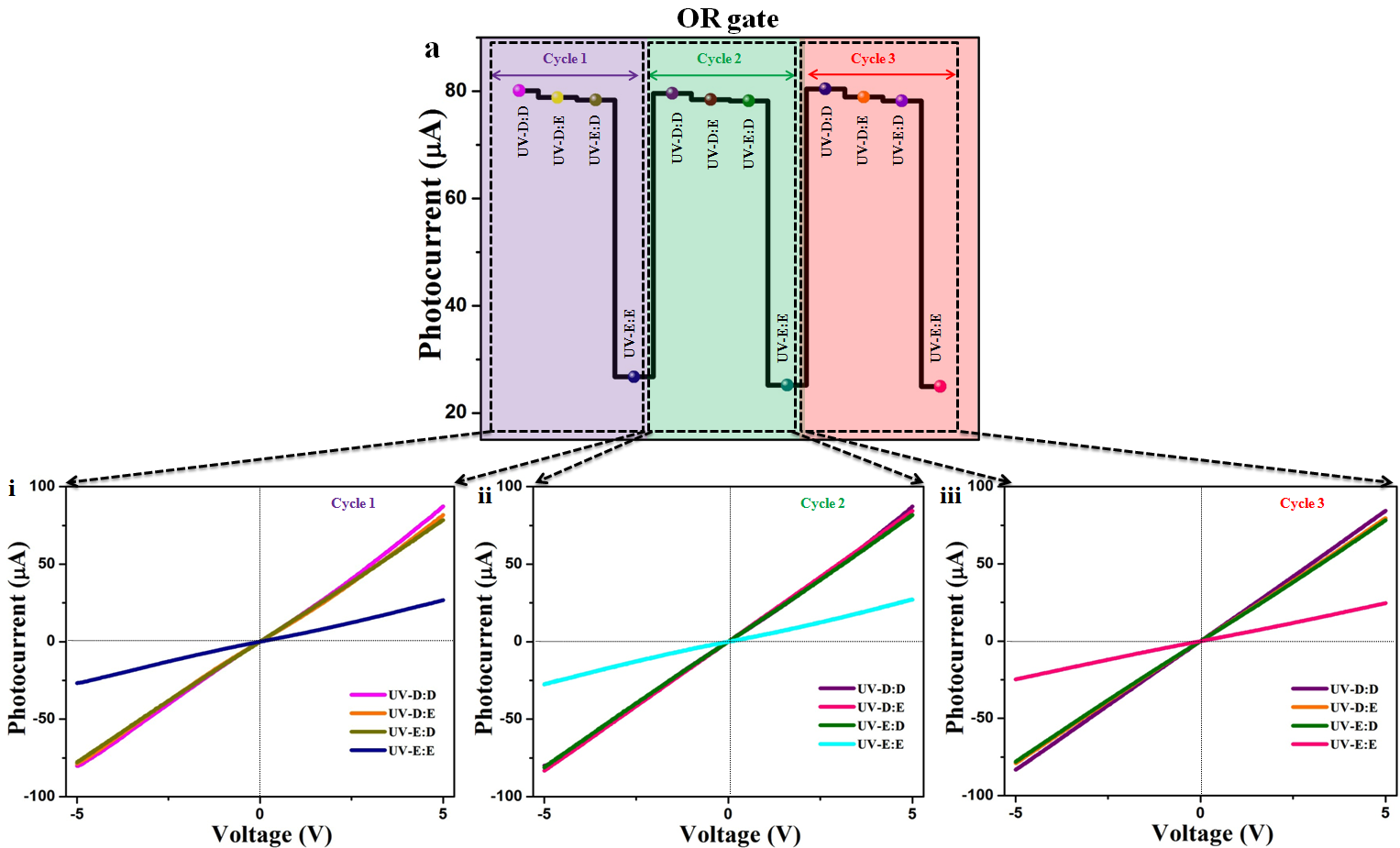
**

**Supplementary Figure 11 a** Photocurrent stability of Y-OF device (D1) operated for three cycles under OR gate conditions. **i – iii** Corresponding I-V plots.

**
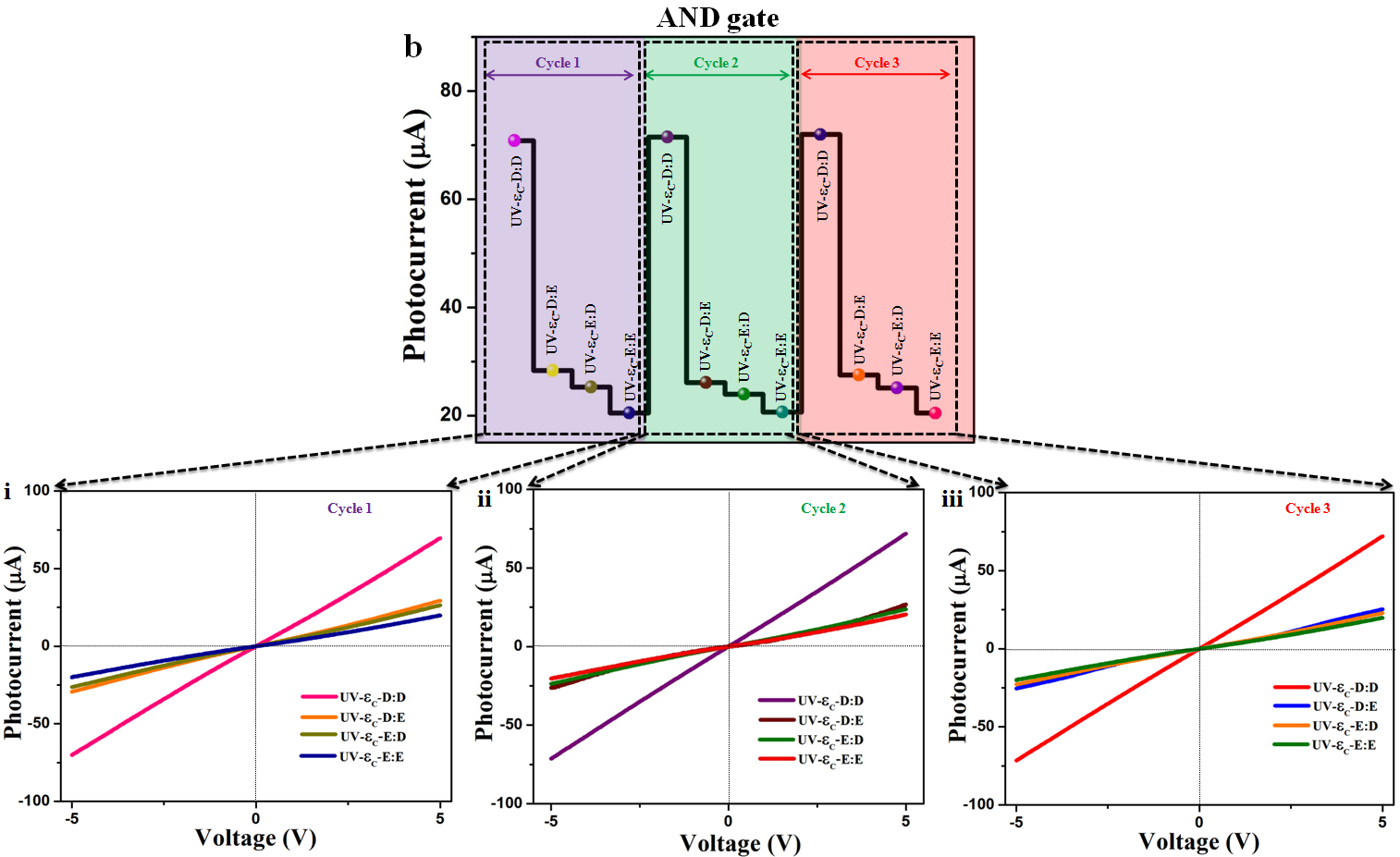
**

**Supplementary Figure 11 b** Photocurrent stability of Y-OF device (D1) operated for three cycles under AND gate conditions. **i – iii** Corresponding I-V plots.

**Penetration depth of light in fluids:**

The evidence on penetration depth (δ_P_) is investigated through UV-Vis absorption spectroscopy which directly measures the penetration capability of light through the liquids^7-9^ given as,

δ_P_ = 1/𝞪 ----------------- (2)

𝞪 = (2.303$\times$A)/t ----------------- (3)

where 𝞪 represents the absorption co-efficient, A is the absorbance and t is the thickness (path length).

Supplementary Figure 12a illustrates the absorbance spectrum of solvents such as ethanol, decanol and ethanol: decanol.

**
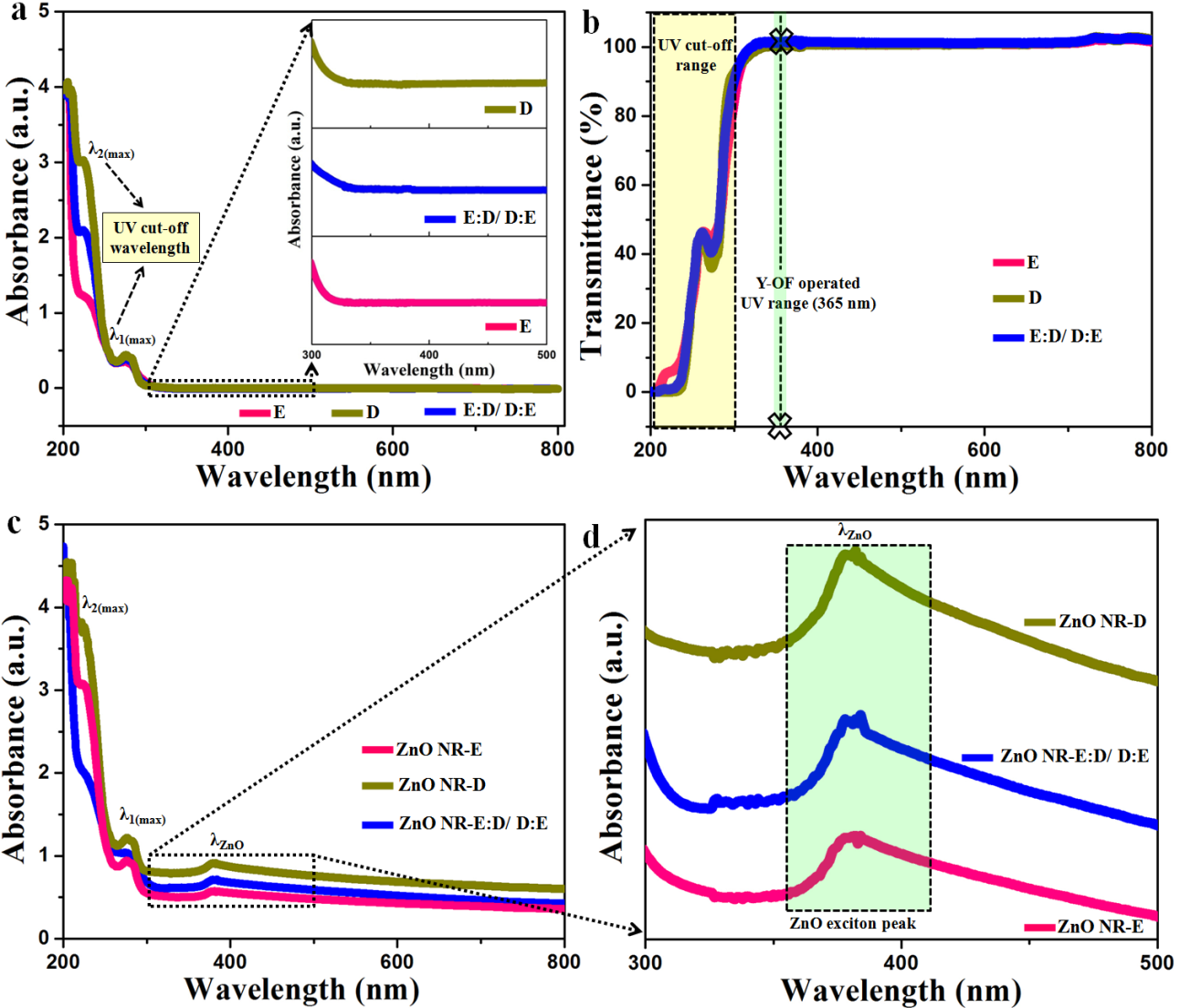
**

**Supplementary Figure 12** UV-Vis spectroscopy analysis of fluids (ethanol (E), decanol (D), and ethanol: decanol (E: D/ D:E)). **a** Absorbance spectrum of fluids without ZnO NR (inset: enlarged view of spectrum in the UV-Vis range, 300-500 nm). **b** Corresponding transmittance plot (without ZnO NR). **c** Absorbance spectrum of fluids in presence of ZnO NR. **d** Enlarged view illustrating the ZnO exciton peak (λ_ZnO_).

As observed, the solvents exhibited an absorbance at a wavelength less than 300 nm which is in accordance with the UV cut-off wavelength of ethanol and decanol. Being aliphatic alcohols, the activation absorption do not fall in the UV-Vis range with the σ-σ* transitions (C-C, C-H); however with possible n-σ* and π-π* transitions (C-O, O-H) the cut-off limit lies in the lower UV range of 200-300 nm^10,11^. Hence, ethanol and decanol absorbs the light at its respective cut-off wavelength (λ_1(max)_, λ_2(max)_) with no significant interference to the UV-Vis ranges >300 nm. Supplementary Figure 12b shows the corresponding spectrum illustrating complete transmittance of the light falling apart its cut-off zone. This confirms that the UV illuminated using LED source (operated at 365 nm) penetrates through the fluidic channel without any hindrance of UV light absorption by the solvents (ethanol and decanol). It assures >99 % transmittance of irradiated 365 nm UV light on ZnO NR. This was further examined through the UV-Vis spectroscopy of solvents (ethanol, decanol, and ethanol: decanol) in presence of ZnO NR (supplementary Figure 12c, d). Along with the absorption cut-off wavelength of solvents, an exciton peak is observed in the UV range of 360-380 nm corresponding to the characteristic peak of hexagonal wurtzite ZnO. Consequently, confirming the irradiation capability of UV light on ZnO NR in the Y-OF device operated for logic conditions.

Therefore, the penetration depths (δ_P_) of light in fluids are further determined from the absorption co-efficient using equations - (2, 3) and the corresponding depth profiles are shown in supplementary Figure 13.

**
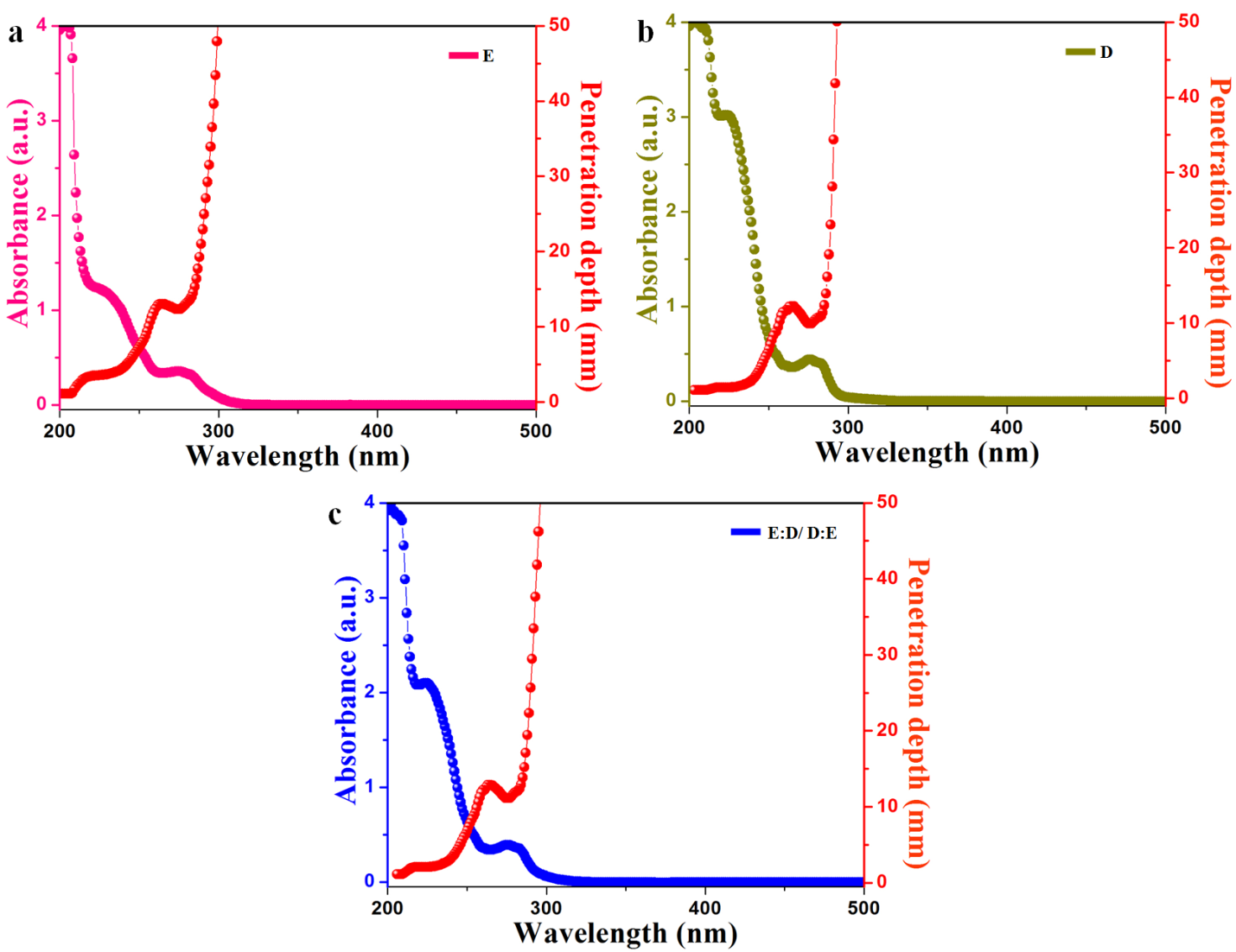
**

**Supplementary Figure 13** Penetration depth in fluids as a function of UV-Vis absorbance spectrum. **a** Ethanol (E). **b** Decanol (D). **c** Ethanol: Decanol (E:D)/ Decanol: Ethanol (D:E).


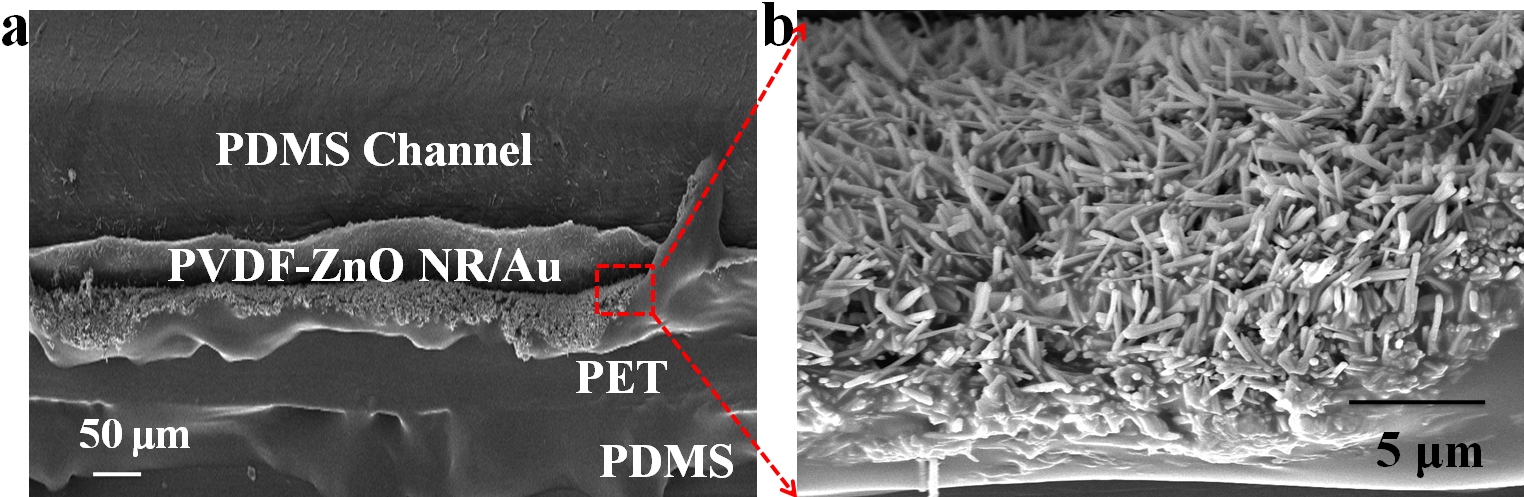


**Supplementary Figure 14** Morphological stability of ZnO NR examined through FESEM analysis after the PPOF experimental studies.

**Choice of VOC as logic operands:**

The suitability of VOC to serve as logic input sources (I/P) is optimized based on the photocurrent factor, where decanol (D) with a high response is fixed for binary ‘1’ and other solvents such as ethanol (E), acetone (A), benzene (B), triethylamine (T), and methanol (M) with reduced response occupy ‘0’ value (Figure 1c). Hence, we test the gate performances with combinations of either ethanol/ acetone/ benzene/ triethylamine/ methanol vs. decanol i.e. the series of possible combinations would be D:E, D:A, D:B, D:T, and D:M.

The I-V behavior of acetone and decanol (D:A) combinations is summarized in supplementary Figure 15a, b. As observed, D:A interacts in the presence of light irradiation (UV-D:A) and lowers the photocurrent to ~ 16.53 μA. In binary terms, UV-D:A condition produces output logic ‘0’ for “1:0” input condition which is not the case for OR operation. As per the concept of strain effects (compressive increases the MSM barrier height and tensile strain reduces the MSM barrier height), we preferred tensile strain ($\varepsilon_{T}$) to switch output ‘0’ to ‘1’ such that D:A works for OR conditions. In spite of the applied strain of +1 %, the fewer improved photocurrent falls within the threshold limit of ‘0’. Thus confirming the combination of D:A is not suitable to perform intrinsic switching of OR$\boldsymbol{\leftrightarrow}$AND gate. Similar behavior was observed for decanol and triethylamine (D:T) proving its unsuitability for intrinsic switching between ‘1’ and ‘0’ logic states (supplementary Figure 15c, d).

**
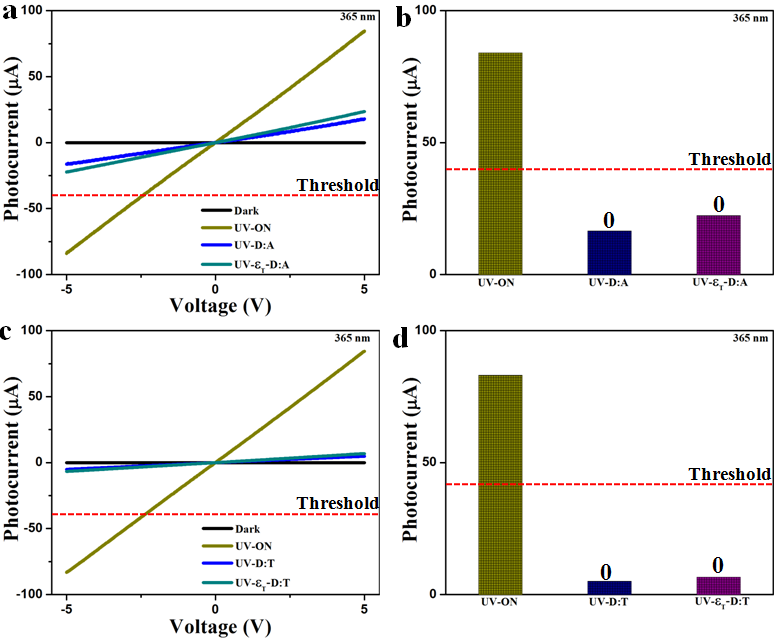
**

**Supplementary Figure 15** **a** I-V curve of decanol and acetone (D:A) under the influence of tensile strain (+1 %). **b** Comparative plot of D:A photocurrent conditions. **c** I-V response of decanol and triethylamine (D:T) at +1 % strain. **d** Corresponding comparison of D:T characteristics.

Besides, when benzene was tested, it reacted with PDMS polymer making the device vulnerable to further investigate the behavior with decanol combination. The PDMS channel crippled when exposed to continuous benzene interaction thus making benzene out of the preferences for further studies. Methanol with least deviation in photocurrent to that of decanol (Figure 2c) is not validated further as it may not show significant differences in switching characteristics. Hence, of the reported VOC in Figure 2c ethanol and decanol (E:D) shows the sensibility to operate OR gate with intrinsic reconfigurable switching nature to AND gate under strain effects.


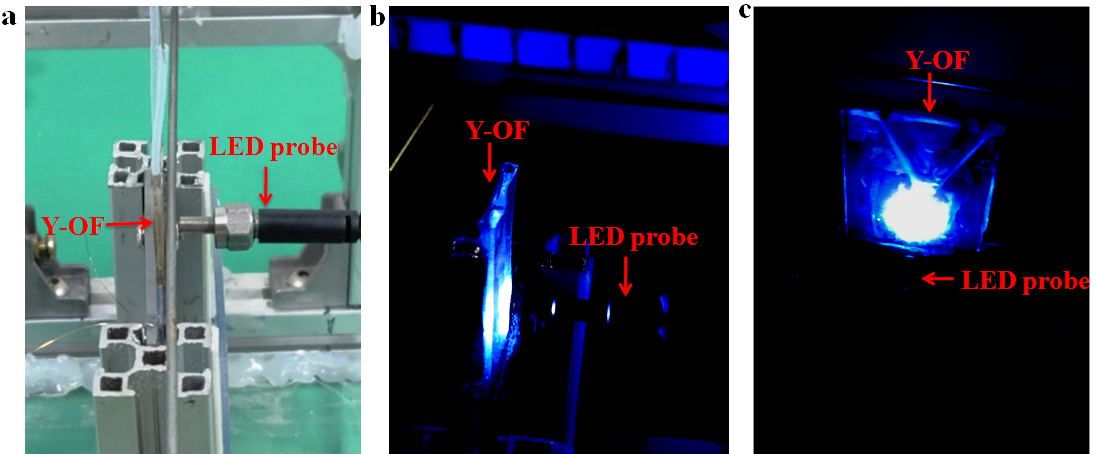


**Supplementary Figure 16** Experimental illustration of Y-OF photoelectric analysis. **a** Under UV-OFF. **b, c** Under UV-ON condition showing homogenous irradiation over the sensing area.


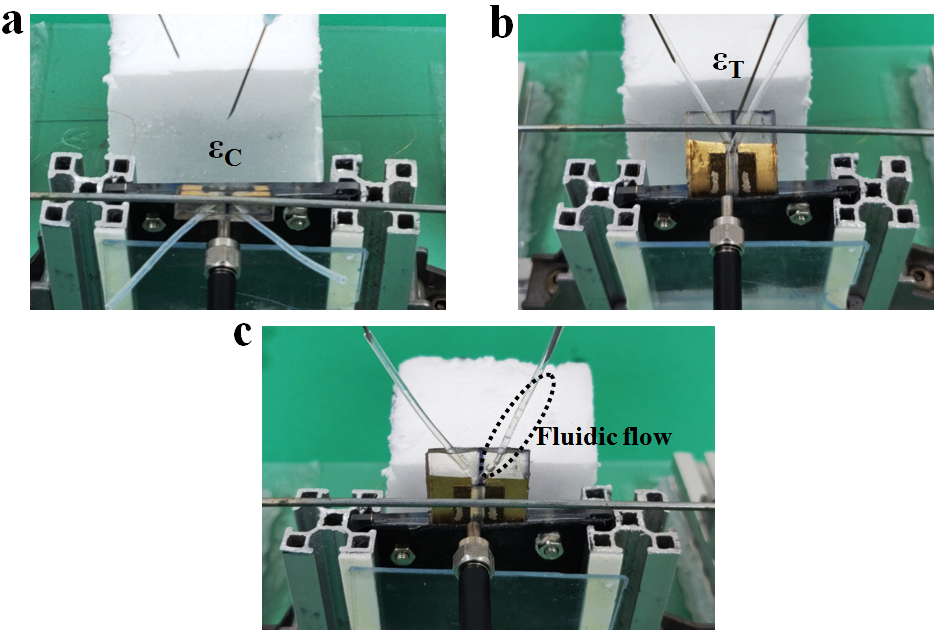


**Supplementary Figure 17** Optical images of Y-OF bending nature through horizontally movable rod (R) determining the strain %.

**Piezoelectric analysis:**

The mechanical force to Y-OF devices are exerted using a linear motor (LinMot-HF01-37) accelerated at 1m/s^2^ with a moving shaft of mass 2 kg (F = 2 N). The piezoelectrical responses are captured using the Keithley electrometer 6514, Keithley Instruments, Cleveland, OH, USA. Supplementary Figure 18 shows the pictorial images of piezoelectric measurement setup used for the experimental analysis.


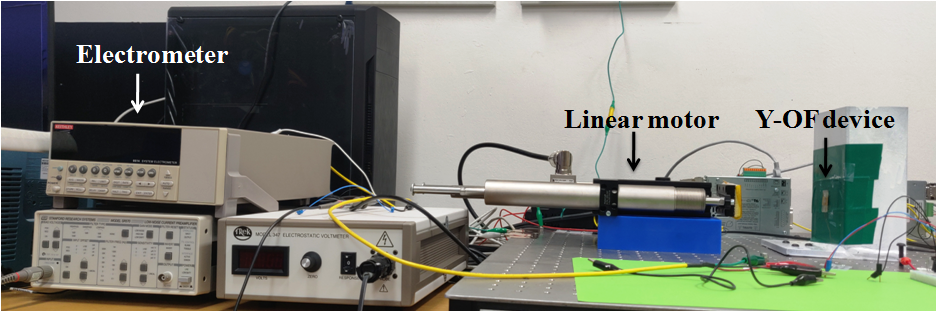


**Supplementary Figure 18** Pictorial image of a piezoelectric measurement unit.


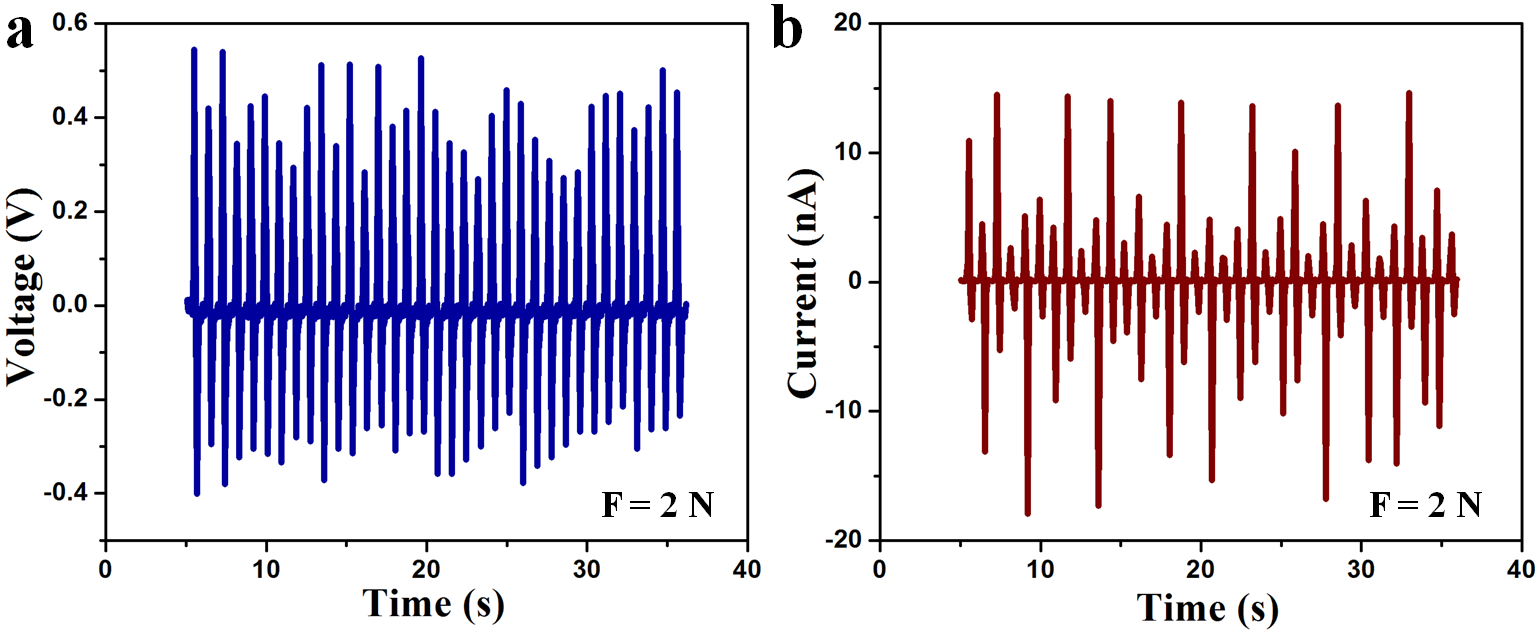


**Supplementary Figure 19** Piezoelectrical analysis under excitation of 2 N forces on Y-OF. **a** Piezoelectric voltage. **b** Piezoelectric current responses.

Further, we demonstrated experiments using the fabricated Y-OF device that could provide the potential real-time illustration of the proposed concept using piezo-phototronic and optofluidic effects referred to self-powered optofluidic logic systems.


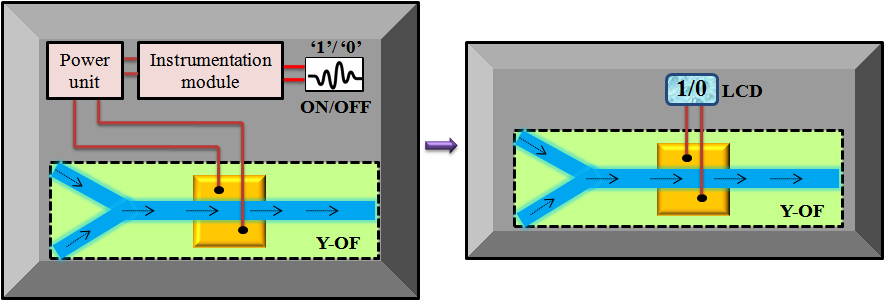


**Supplementary Figure 20** Simplified overview of proposed concept to the self-powered system.

An overview of a simplified self-powered dimension is schematically illustrated in supplementary Figure 20. As a point of proof to validate the practicality of the proposed system, we have shown the self-powered mode of Y-OF device, and the switching is directly read through the display (LCD). This mode is enabled through applying a constant force of 2 N to the Y-OF by utilizing the generated piezoelectric potentials (supplementary Figure 19) to drive the LCD (and other low power electronics) without using an external power supply/bias voltage. Supplementary Figure 21 and supplementary Video 1 show the real-time analysis of AND gate in self-powered Y-OF, which is modulated through the interfacial effects of force, light, and fluids. AND gate is demonstrated with maintaining a constant force and light source whereby changing the fluidic input between decanol and ethanol. The same operations are followed as illustrated in manuscript to perform AND gate (i.e., UV-$\varepsilon_{C}$-D:D, UV-$\varepsilon_{C}$-E:D, UV-$\varepsilon_{C}$-D:E and UV-$\varepsilon_{C}$-E:E). Here the strain factor ($\varepsilon_{C}$) refers to force applied to the device, and we tested using two syringes (S_1_, S_2_) since this is to analyze the effect of decanol and ethanol. In the presence of UV-force-decanol, the LCD is ON reading to the AND logic output ‘1’. In the case of UV-force-decanol: ethanol and UV-force-ethanol, the LCD is turned OFF, which can be related to the AND logic output ‘0’. This shows the possibility to realize the three metrics such as piezoelectric-photoelectric-fluid gating to have a potential practical realization. To perform multiple logic gates and its intrinsic switching, the tunable parameter has to be examined in the near future based on the flexibility of metrics analyzed.

**
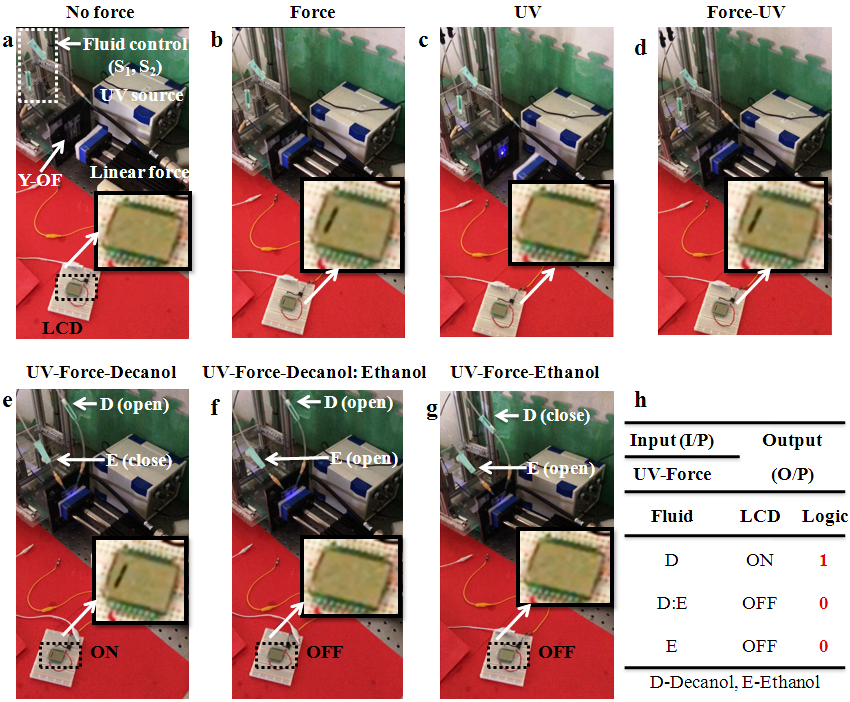
**

**Supplementary Figure 21** Practical realization of piezo-phototronic gated optical switching (self-powered mode) using S_1_ and S_2_ as fluid inlets. **a-d** UV-Force conditions. **e-g** UV-Force-Fluid conditions. **h** Summarization of observed results referring to AND logic gate.

**Supplementary References**

1. Zou, Y., Namkung, J. et.al. Influence of a bias voltage on surface-driven orientational transitions for liquid crystal-based chemical and biological sensors. *J. Phys. D: Appl. Phys.* **44**, 135103 (2011).
2. Purusothaman, Y., Alluri, N. R. et al. Regulation of Charge Carrier Dynamics in ZnO Microarchitecture-Based UV/Visible Photodetector via Photonic-Strain Induced Effects. *Small* **14**, 1703044 (2018).
3. [Liu, Y.,](https://www.sciencedirect.com/science/article/pii/S2211285514002596#!) Zhang, Y. et al. Fundamental theories of piezotronics and piezo-phototronics. [*Nano Energy*](https://www.sciencedirect.com/science/journal/22112855) **14**, 257–275 (2015).
4. Mandalapu, L. J., Xiu, X. F. et.al p-type behavior from Sb-doped ZnO heterojunction photodiodes. *Appl. Phys. Lett.* **88**, 112108 (2006).
5. Janotti. A., Van de Walle, G, C. Oxygen vacancies in ZnO. *App. Phys. Lett*. **87**, 122102 (2005).
6. Djurisic, A., Chen, X. et.al. ZnO nanostructures: growth, properties and applications. ***J. Mater. Chem.*,** **22**, 6526-6535 (2012).
7. Liu, Z., Zhou, C. et.al. [Quantifying the concentration and penetration depth of long](https://aip.scitation.org/doi/full/10.1063/1.5037660)-[lived RONS in plasma](https://aip.scitation.org/doi/full/10.1063/1.5037660)-[activated water by UV absorption spectroscopy](https://aip.scitation.org/doi/full/10.1063/1.5037660). [*AIP Advances*](https://aip.scitation.org/doi/full/10.1063/1.5037660) **9**, 015014 (2019).
8. Tokudome, Y., Suzuki, K., Kitanaga, T., Takahashi, M. Hierarchical Nested Wrinkles on Silica2Polymer Hybrid Films: Stimuli-Responsive Micro Periodic Surface Architectures. *Scientific Reports* **2**, 683 (2012).
9. Graaf, G., Wolffenbuttel, F. R. Illumination Source Identification Using a CMOS Optical Microsystem. *IEEE T. Instrum. Meas.* **53**, 238-242 (2004).
10. Räty, A. J., Peiponen, K. E., Asakura, T. UV-Visible Reflection Spectroscopy of Liquids: Springer Series in Optical Sciences. *ISBN 978-3-540-45093-1* (2004).
11. Katz, E., Eksteen, R., Schoenmakers, P., Miller, N. Handbook of HPLC: Chromatographic Science Series. *ISBN 0-8247-9444-3* (2000).
