# Supplementary material for: Piezophototronic gated optofluidic logic computations empowering intrinsic reconfigurable switches
Source: Nat Commun. 2019 Sep 26;10:4381. doi: 10.1038/s41467-019-12148-y (PMC6763476; doi:10.1038/s41467-019-12148-y)
Supplement: Supplementary file 2 — Description of Additional Supplementary Files [file 41467_2019_12148_MOESM2_ESM.pdf]

Description of Additional Supplementary Files

File name: Supplementary Movie 1

Description: Demonstration of LCD (ON/OFF) using the logic switching behavior of Y-OF (AND gate)
